# Supplementary material for: Capsules and their traits shape phage susceptibility and plasmid conjugation efficiency
Source: Nat Commun. 2024 Mar 6;15:2032. doi: 10.1038/s41467-024-46147-5 (PMC10918111; doi:10.1038/s41467-024-46147-5)
Supplement: Supplementary file 1 — Supplementary Information [file 41467_2024_46147_MOESM1_ESM.pdf]

# Capsules and their traits shape phage susceptibility and plasmid conjugation efficiency

*Matthieu Haudiquet[1,2*], Julie Le Bris[1,3], Amandine Nucci[1], Rémy A. Bonnin[4], Pilar Domingo-Calap[5], Eduardo P.C. Rocha[1†], Olaya Rendueles[1†]*

## Supplementary Figures

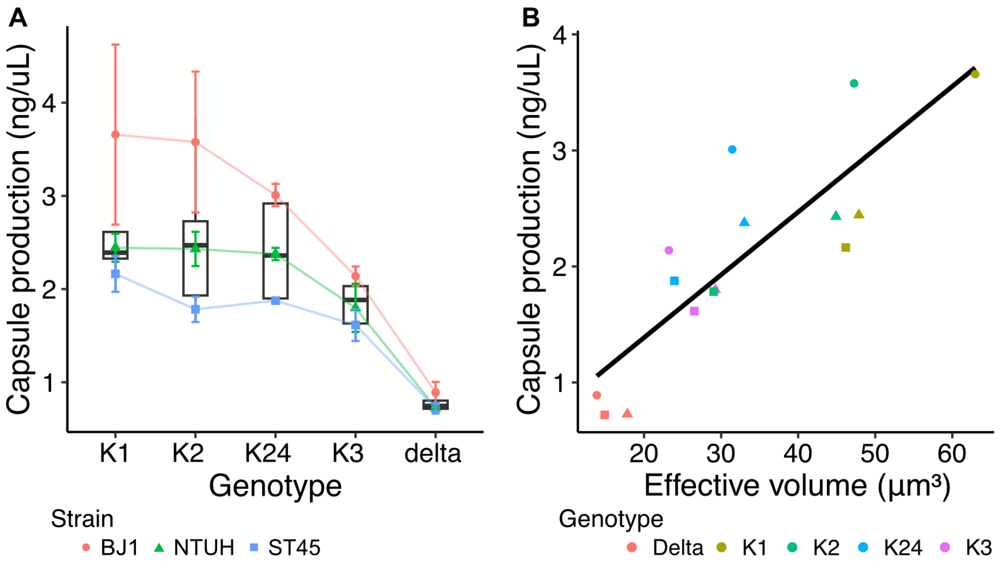


**Supplementary Figure S1 – Capsule quantification. A.** Capsule production estimated by glucuronic acid dosage in capsule extracts (See *Glucuronic acid assay*). Each point represents the mean of independent replicates (N=3), point shapes represent the strain chassis, and error bars represent the standard deviation. Box plots are drawn from all observations (N=9) for each genotype. B. Capsule production in function of the effective volume. Each point represents the mean of independent triplicates. Shapes correspond to the strain chassis as in panel A, colors correspond to the genotype. The linear mixed model of capsule production as a response variable, effective volume and chassis strain as random effect gives a significant effect of the volume (F-test, $p<0.001$) and an $R^2=0.8$ (Statistics 6e). Associated data are available as Source Data 6 and Source Data 11.

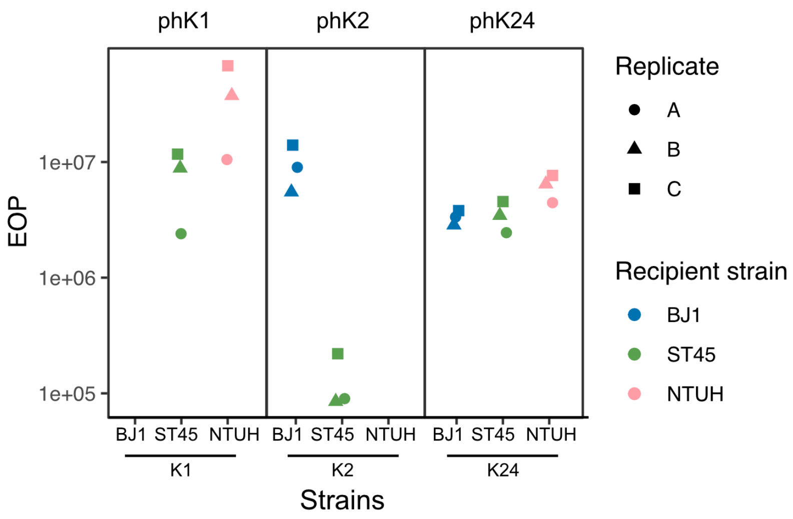


**Supplementary Figure S2 – Phage infection assay.** Raw values of efficiency of plating (EOP) for each swapped strain. Replicates A, B and C correspond to independent phage lysates prepared with the wildtype strains. phK1 against ST45::K2 and phK2 against NTUH::K2 do not result in productive infections. Associated data are available as Source Data 2.

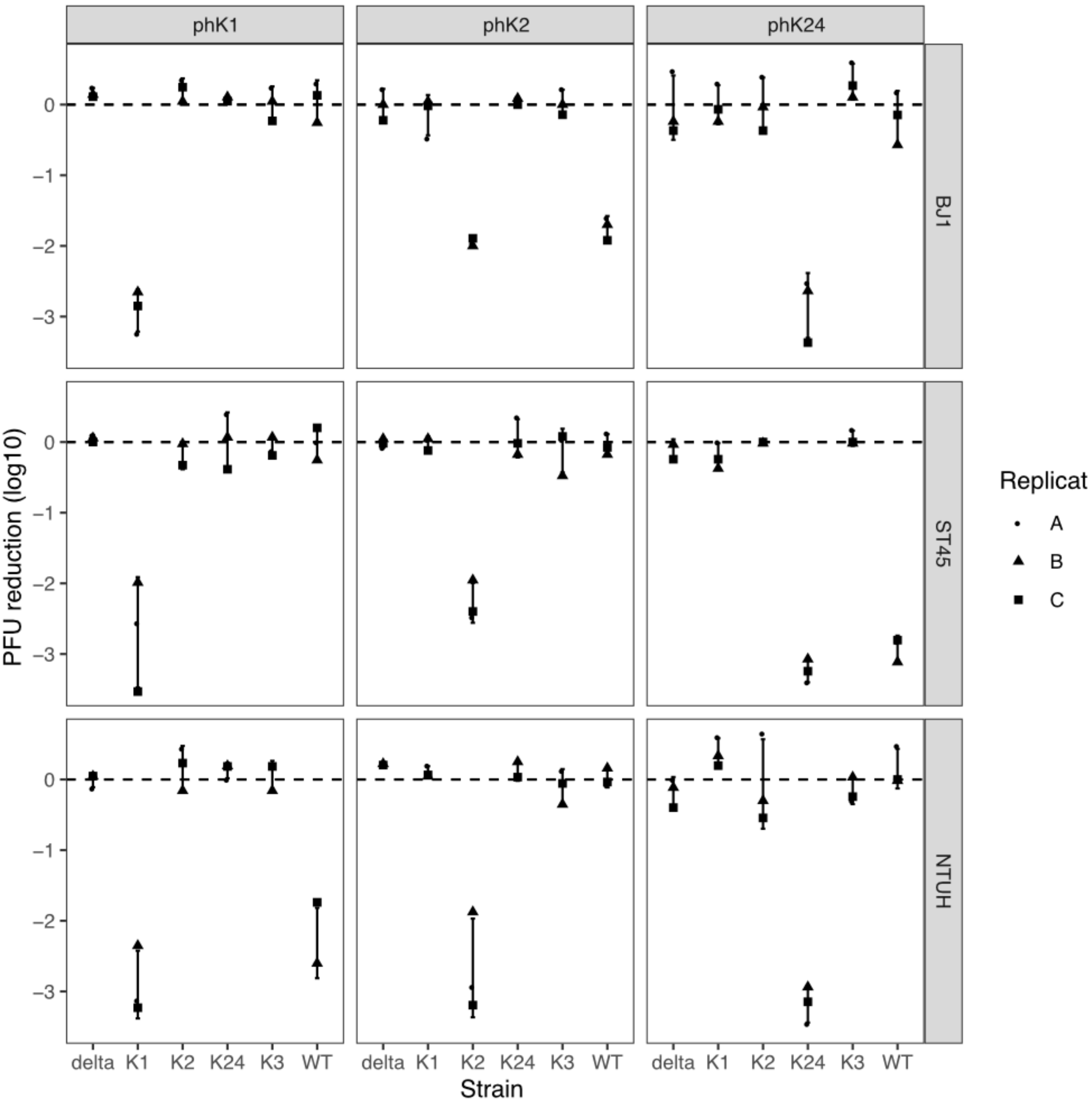


**Supplementary Figure S3 – Adsorption assay.** Phage adsorption onto the different strains. Shapes correspond to independent biological replicates. Adsorption is quantified as the $\log_{10}$-transformed relative PFU reduction after 5 minutes of incubation. A value of 0 indicates no adsorption. Associated data are available as Source Data 1.

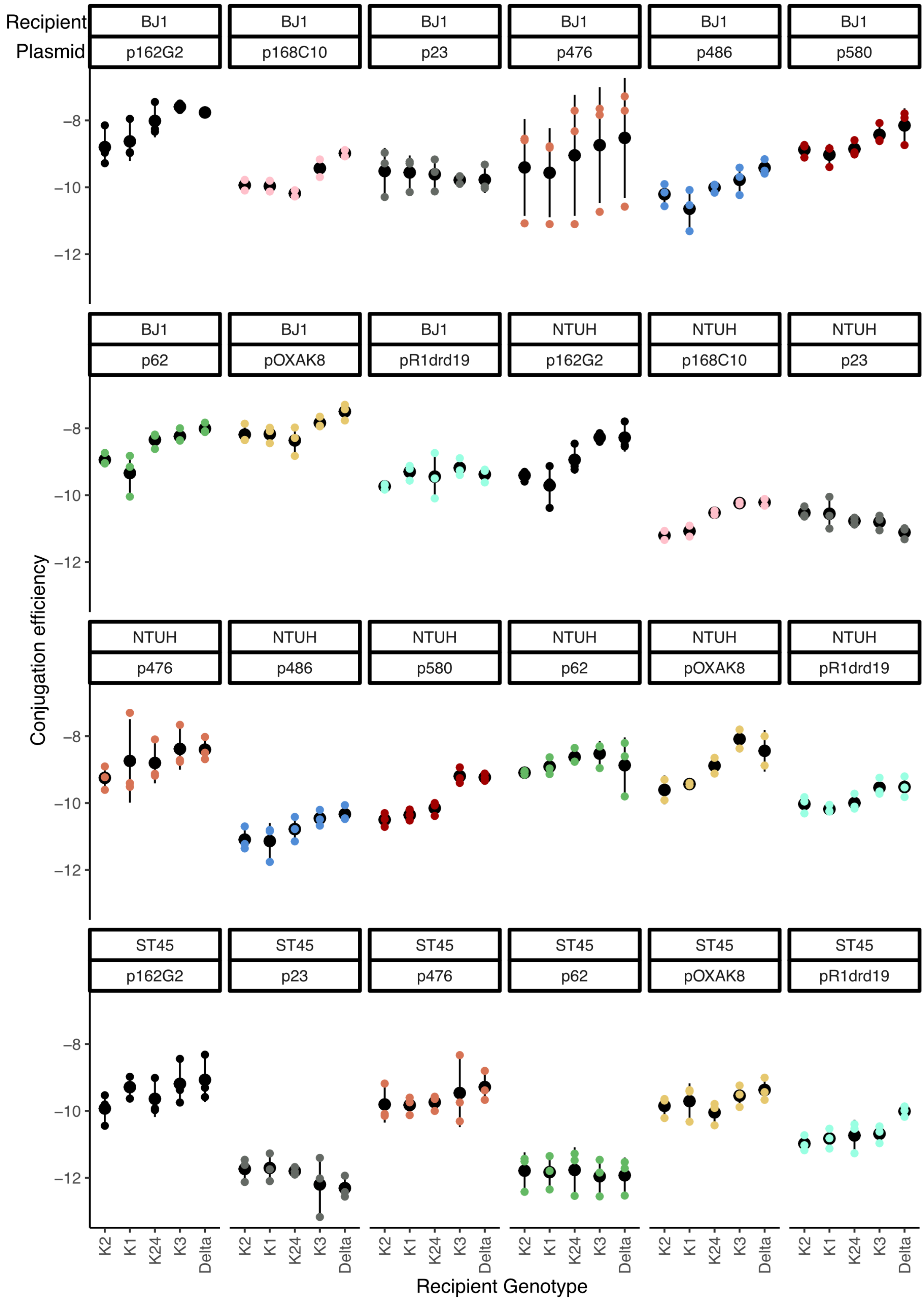


**Supplementary Figure S4 – Individual conjugation assays E1.** Log-transformed conjugation efficiency in the E1 dataset from an *E. coli* donor according to the genotype of the recipient strain. Plots are faceted by the donor strain and plasmid. Coloured points represent independent biological replicates,

black points represent their average (mean) and error bars the standard deviation. Associated data are available as Source Data 3.

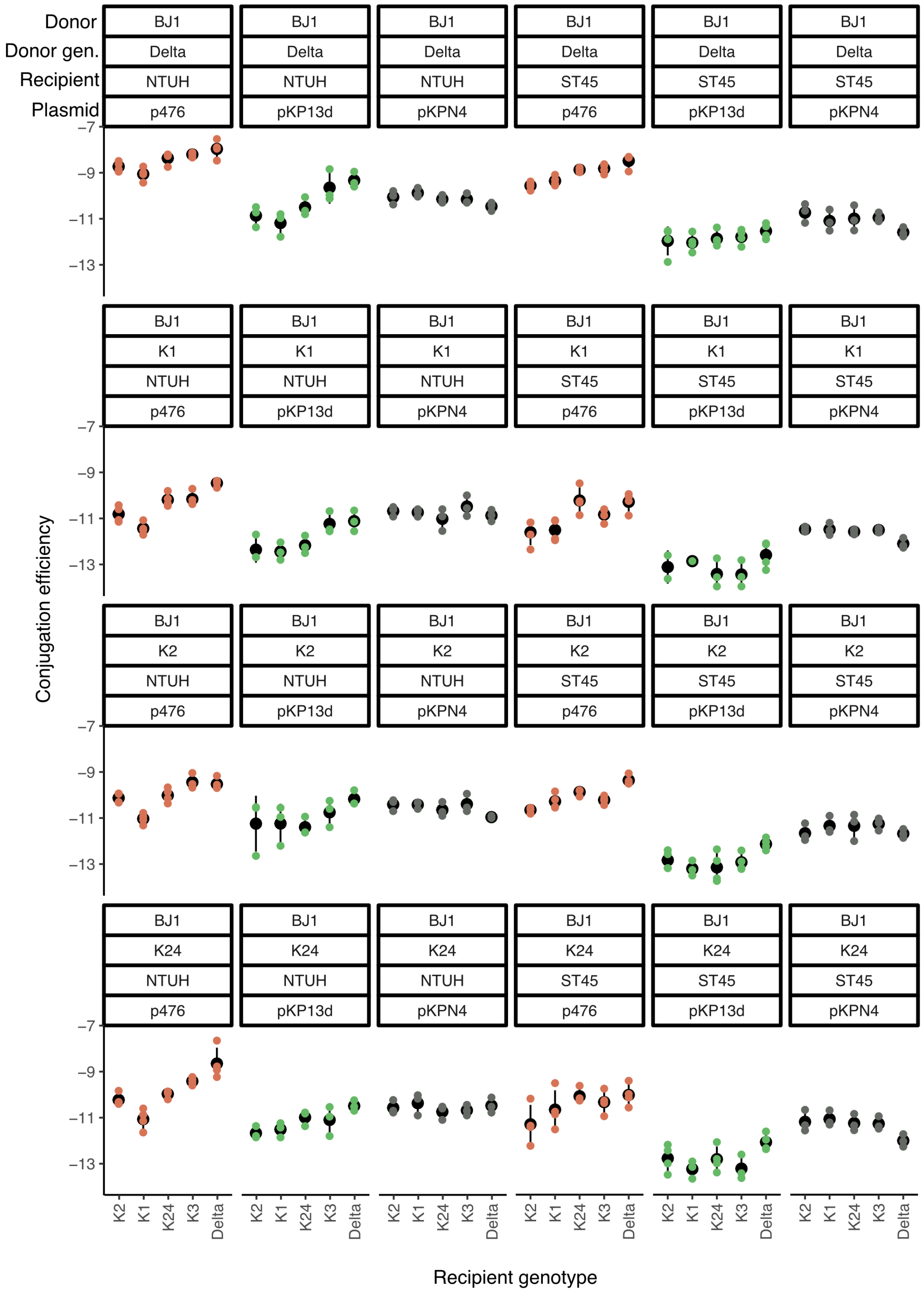
Donor
Donor gen.
Recipient
Plasmid
BJ1
Delta
K1
K2
K24
NTUH
ST45
p476
pKP13d
pKPN4
−7
−9
−11
−13
Conjugation efficiency
K2
K1
K24
K3
Delta
Recipient genotype

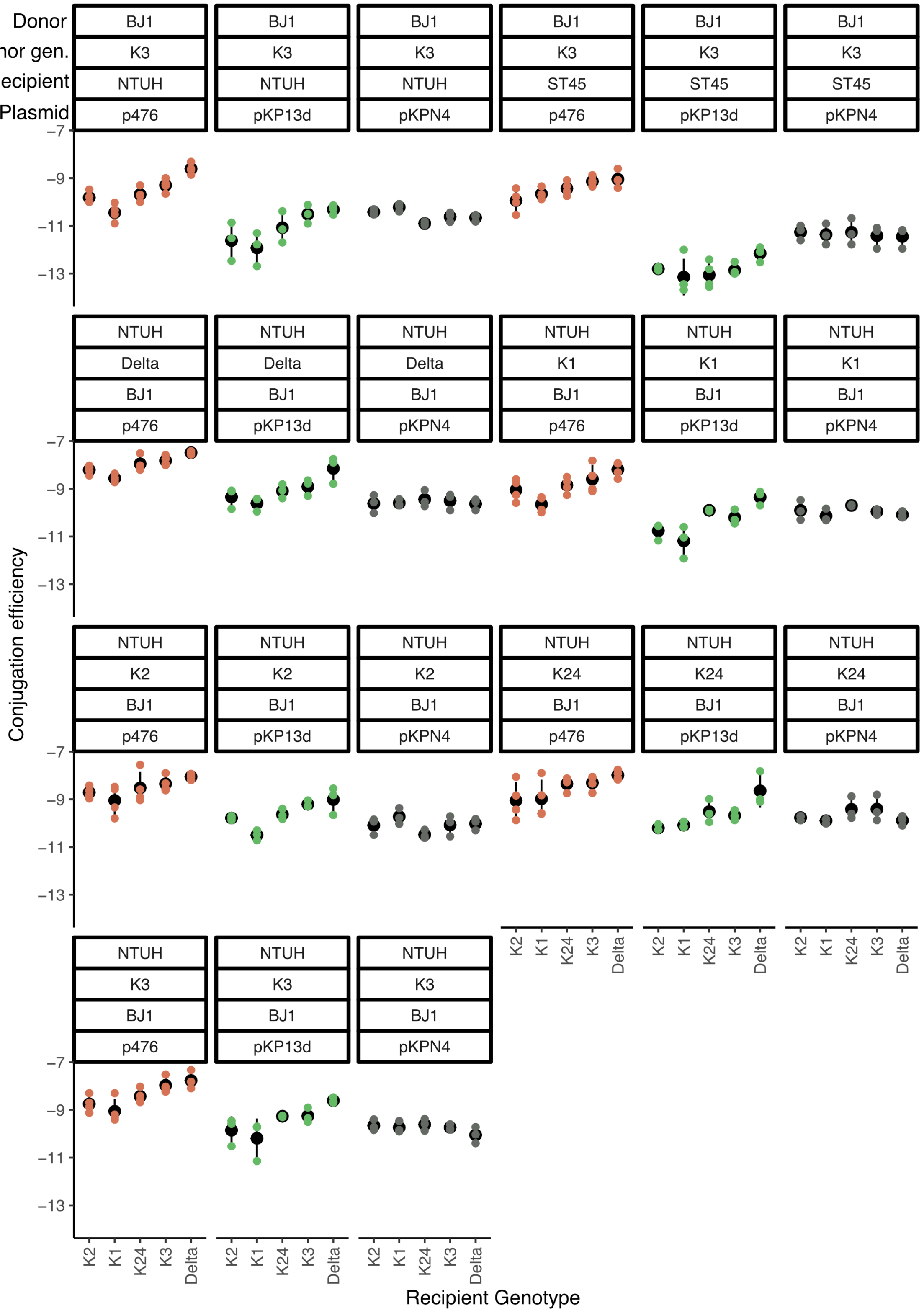


**Supplementary Figure S5 – Individual conjugation assays E2.** Log-transformed conjugation efficiency in the E2 dataset from *K. pneumoniae* donors according to the genotype of the recipient strain. Plots are faceted by the donor strain, donor genotype, recipient strain and plasmid. Colored points

represent independent biological replicates, black points represent their average (mean) and error bars the standard deviation.
Associated data are available as Source Data 4.

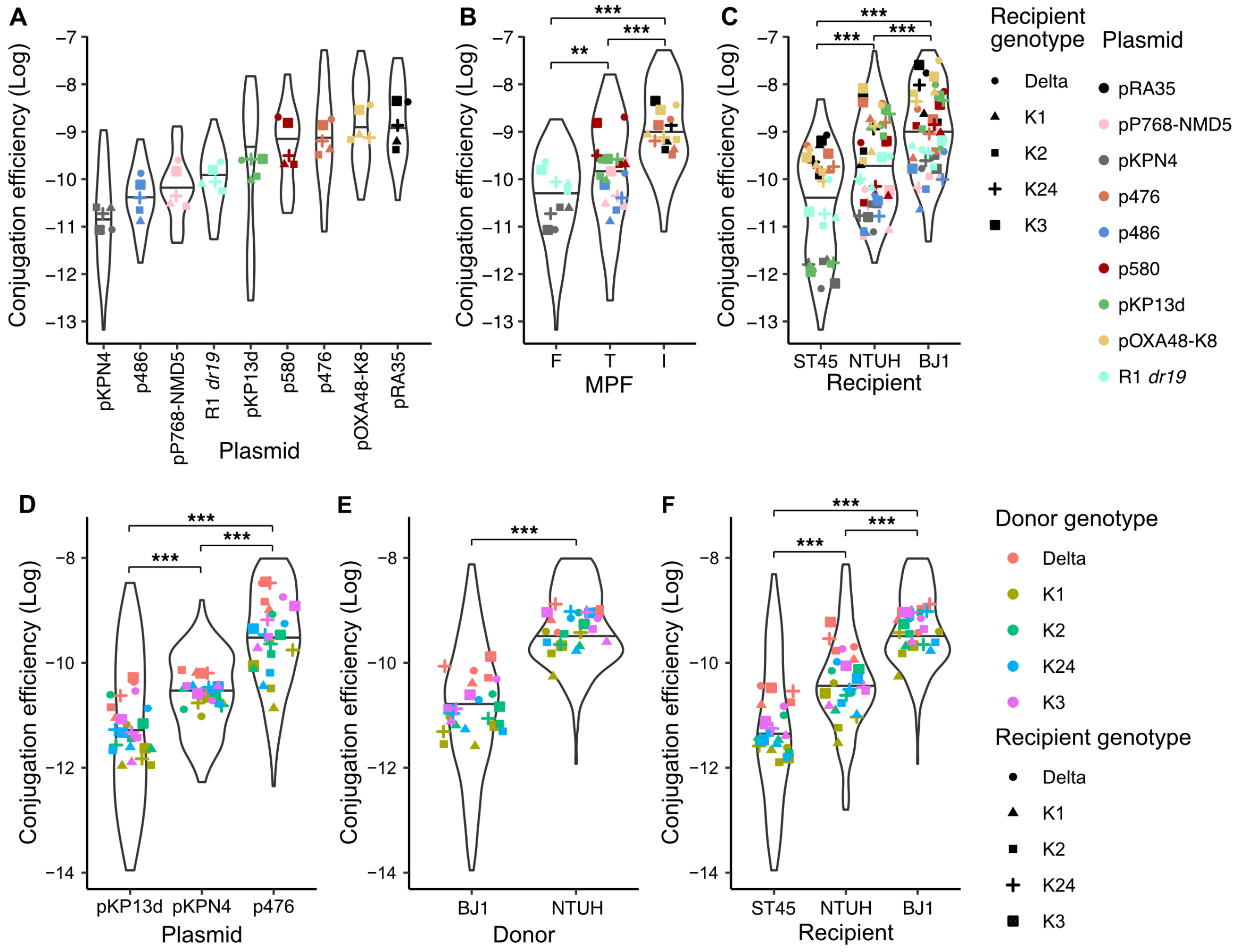


**Supplementary Figure S6 – Factors accounted for in the multi-variate analysis.**
**A, B, C.** *E. coli* to *K. pneumoniae* assays (set E1).
**D, E, F.** *K. pneumoniae* to *K. pneumoniae* assays (set E2).

A. Log-transformed conjugation efficiency of each plasmid. Individual points are coloured according to the plasmid and represent the average of each recipient genotype (shape).
B. Same data as in A, according to the different MPF types of the plasmids (x-axis).
C. Log-transformed conjugation efficiency of each plasmid (colour) according to the recipient genotype (shape) and recipient strain (x-axis).
D. Log-transformed conjugation efficiency for each plasmid (x-axis) according to the donor (colour) and recipient (shape) genotype.
E. Same data, according to the different donor strains (x-axis).
F. Same data, according to the different recipient strains (x-axis).

Statistical tests: Pairwise Wilcoxon tests. *** $p$<0.001; ** $p$<0.01
Associated data are available as Source Data 3 and Source Data 4.

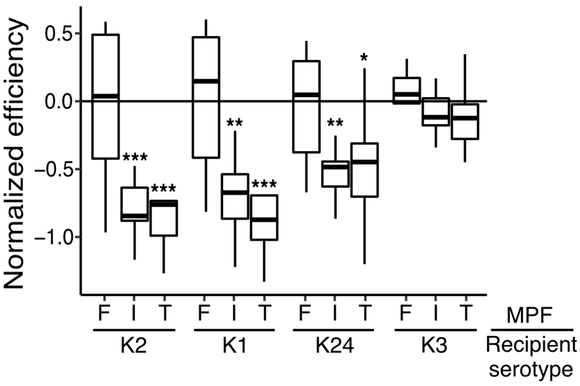


**Supplementary Figure S7 – Conjugation efficiency of MPF types in distinct recipient serotype**
Drawn from the *E. coli* to *K. pneumoniae* assays (Set E1). The normalized efficiency corresponds to the log-transformed conjugation efficiency of each plasmid-recipient pair subtracted with the one of the corresponding Δcps mutant:

$$Normalized\ efficiency_{Plasmid-Recipient} = \log\left(Conj.efficiency_{\mathrm{cps+}}\right) - \log\left(Conj.efficiency_{\Delta\mathrm{cps}}\right)$$

Below the solid line at 0, the capsulated cells have a lower conjugation efficiency than the Δcps. At 0, there is no difference with the Δcps. Above 0, the capsulated cells have higher conjugation efficiency than the Δcps.
Statistical tests: Wilcoxon test (H0: average =0). *** $p$<0.001; ** $p$<0.01; * $p$<0.05
Associated data are available as Source Data 3.

```
OmpK36-BJ1      MKVKVLSLLVPALLVAGAANAAEIYNKDGNKLDLYGKIDGLHYFSDDKSVDGDQTYMRVG    60
OmpK36-NTUH     MKVKVLSLLVPALLVAGAANAAEIYNKDGNKLDLYGKIDGLHYFSDDKSVDGDQTYMRVG    60
OmpK36-ST45     MKVKVLSLLVPALLVAGAANAAEIYNKDGNKLDLYGKIDGLHYFSDDKSVDGDQTYMRVG    60
                ************************************************************

OmpK36-BJ1      VKGETQINDQLTGYGQWEYNVQANNTESSSDQAWTRLAFAGLKFGDAGSFDYGRNYGVVY    120
OmpK36-NTUH     VKGETQINDQLTGYGQWEYNVQANNTESSSDQAWTRLAFAGLKFGDAGSFDYGRNYGVVY    120
OmpK36-ST45     VKGETQINDQLTGYGQWEYNVQANNTESSSDQAWTRLAFAGLKFGDAGSFDYGRNYGVVY    120
                ************************************************************
                             L3 LOOP
OmpK36-BJ1      DVTSWTDVLPEFGGDTYGSDNFLQSRANGVATYRNSDFFGLVDGLNFALQYQGKNGSVSG    180
OmpK36-NTUH     DVTSWTDVLPEFGGDTYGSDNFLQSRANGVATYRNSDFFGLVDGLNFALQYQGKNGSVSG    180
OmpK36-ST45     DVTSWTDVLPEFGGDTYGSDNFLQSRANGVATYRNSDFFGLVDGLNFALQYQGKNGSPSG    180
                ********************************************************* **
                    L4 LOOP
OmpK36-BJ1      EGA---TNNGRGWSKQNGDGFGTSLTYDIWDGISAGFAYSHSKRTDEQNSVPALGRGDNA    237
OmpK36-NTUH     EGA---TNNGRGWSKQNGDGFGTSLTYDIWDGISAGFAYSHSKRTDEQNSVPALGRGDNA    237
OmpK36-ST45     EGALSPTNNGRTALKQNGDGYGTSLTYDIYDGISAGFAYSNSKRLGDQNSKLALGRGDNA    240
                ***   *****   ******:********:**********:*** .:***  ********

OmpK36-BJ1      ETYTGGLKYDANNIYLASQYTQTYNATRAGSLGFANKAQNFEVVAQYQFDFGLRPSVAYL    297
OmpK36-NTUH     ETYTGGLKYDANNIYLASQYTQTYNATRAGSLGFANKAQNFEVVAQYQFDFGLRPSVAYL    297
OmpK36-ST45     ETYTGGLKYDANNIYLATQYTQTYNATRAGSLGFANKAQNFEVVAQYQFDFGLRPSVAYL    300
                *****************:******************************************

OmpK36-BJ1      QSKGKDLERGYGDQDILKYVDVGATYYFNKNMSTYVDYKINLLDDNSFTRNAGISTDDVV    357
OmpK36-NTUH     QSKGKDLERGYGDQDILKYVDVGATYYFNKNMSTYVDYKINLLDDNSFTRNAGISTDDVV    357
OmpK36-ST45     QSKGKDLE-GYGDQDILKYVDVGATYYFNKNMSTYVDYKINLLDDNSFTHNAGISTDDVV    359
                ******** ****************************************:**********

OmpK36-BJ1      ALGLVYQF 365
OmpK36-NTUH     ALGLVYQF 365
OmpK36-ST45     ALGLVYQF 367
                ********
```

**Supplementary Figure S8 – Alignment of OmpK36 proteins from the three *K. pneumoniae* strains.** Proteins aligned with Clustal Omega (https://www.ebi.ac.uk/Tools/msa/clustalo/).

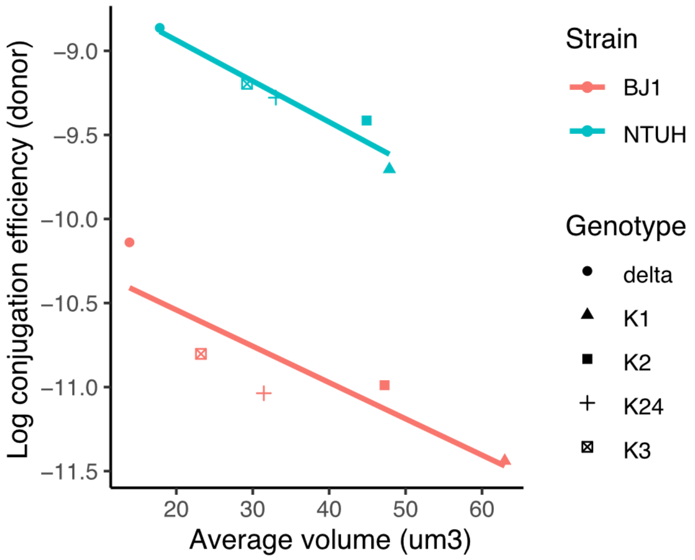


**Supplementary Figure S9 – Donor conjugation efficiency negatively correlates with capsule size.** The average effective volume vs. average $log_{10}$-transformed conjugation efficiencies of donors (from E2) is presented. Points correspond to distinct capsule states in the recipient strain. Colour corresponds to the two *K. pneumoniae* strains used as donor. Lines represent linear regressions for each chassis strain between the $log_{10}$-transformed conjugation efficiency and the average effective volume (EV). The linear mixed model of the $log_{10}$ transformed conjugation efficiency using the effective volume as a fixed effect and the chassis strain identity as a random effect showed a significant effect of the volume (F test, $p < 0.001$). Source data are available as Source Data file 12.

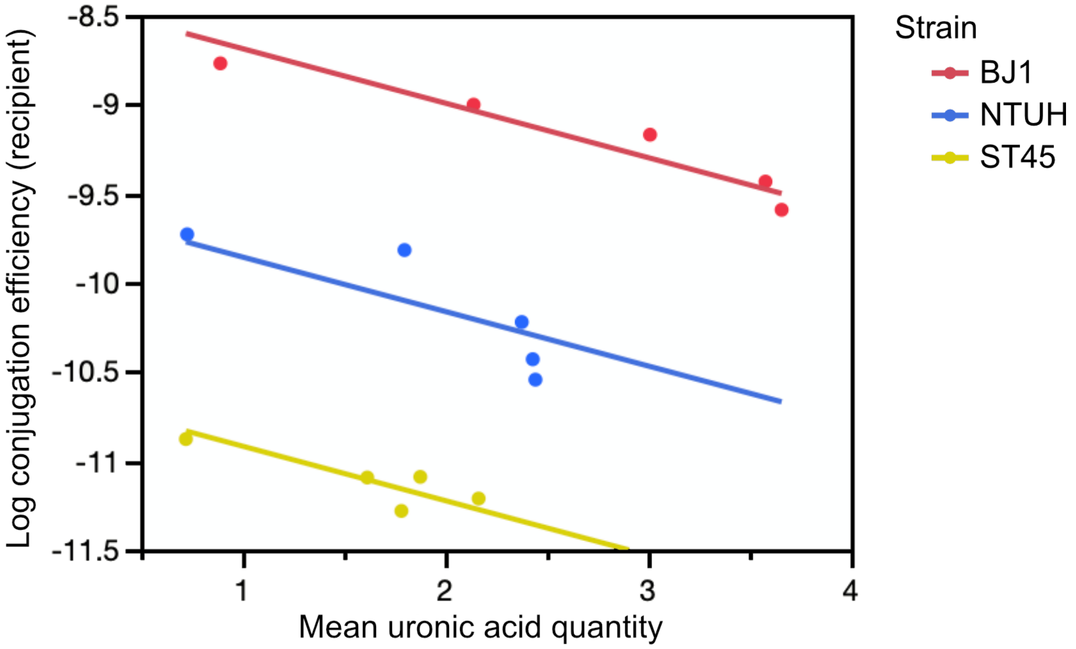


**Supplementary Figure S10 – Recipient conjugation efficiency negatively correlates with uronic acid quantity.** Mean uronic acid quantity vs. mean $log_{10}$-transformed conjugation efficiencies of recipients (from E1 and E2 combined). Points correspond to distinct capsule states in the recipient strain. Colour corresponds to the strains as in panel A. Lines represent linear regressions for each chassis strain between the mean $log_{10}$-transformed conjugation efficiency and the mean uronic acid quantity. We fitted a linear mixed model with the capsule quantification as a fixed effect, the chassis strain as a random effect, and the conjugation efficiency as the response variable. See Statistics 6b and 6d in Supplementary Text S2 ($R2=0.97$, $p<0.001$). Associated data are available as Source Data 10.

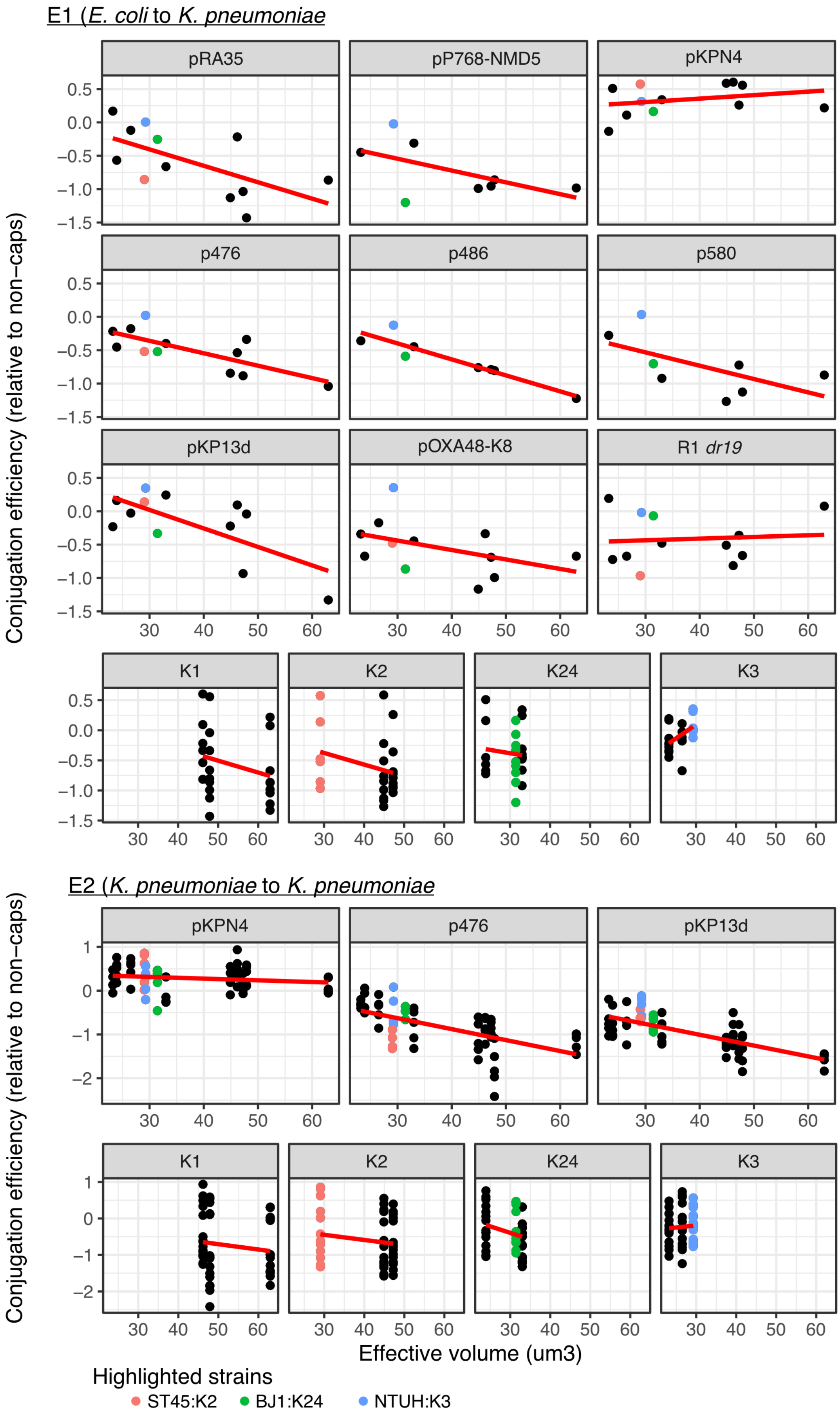


**Supplementary Figure S11 – Conjugation efficiency vs. effective volume.** The conjugation efficiency normalized by the associated non-capsulated mutants from E1 and E2, relative to the effective volume of recipient strains. Plots are facetted according to the plasmid (top rows) and recipient serotype (bottom rows) for E1 and E2. Highlighted strains have very similar volumes but correspond to different strains and serotype. Points are the mean of three biological replicates. Individual linear regressions are represented by red lines. Associated data are available as Source Data 13.

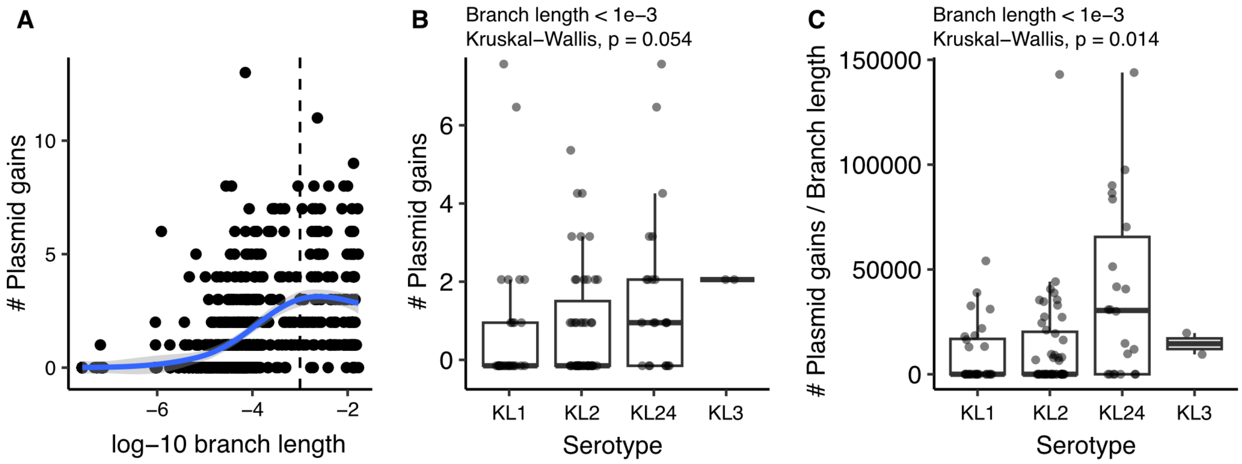


**Supplementary Figure S12 – Plasmid gains according to branches length. A.** Observed plasmid gains vs. branch length ($log_{10}$-transformed). The blue line is fitted by GAM (ggplot2 geom_smooth(method = "gam")) and shows the saturation of plasmid gains above length>0.001 (two outliers long branches >0.01 were removed). **B.** Similar analysis as figure 5B, but with branch length<0.001. Number of plasmids recently acquired (terminal branches of the species tree) in different serotypes. Individual points represent distinct genomes. **C.** Number of plasmids recently acquired divided by the branch length of the strain harbouring them in different serotypes. Only branches <0.001 are considered. Individual points represent distinct genomes. Associated data are available as Source Data 14.

# Supplementary Tables

| Name | Sequence | Usage | Template |
|---|---|---|---|
| **Delcaps_1.F** | TGCCGGATATCATCCTTGAC | Deletion cassette construction | BJ1 genomic DNA (galF) |
| **Delcaps_1.R** | AGCCTACACATCAGGCCAGCAGTTTTTTAATG | Deletion cassette construction | BJ1 genomic DNA (galF) |
| **Delcaps_2.F** | CTGGCCTGATGTGTAGGCTGGAGCTGC | Deletion cassette construction | KmFRT cassette |
| **Delcaps_2.R** | GTAGGGATAACAGGGTAATGacggaagcaaatcatctgca | Deletion cassette construction | KmFRT cassette |
| **Delcaps_3.F** | cgtCATTACCCTGTTATCCCTACaTGAAAATTACTATTTCCGGTACAGG | Deletion cassette construction | BJ1 genomic DNA (ugd) |
| **Delcaps_3.R** | AGGTTGTCGTACAGCGCACG | Deletion cassette construction | BJ1 genomic DNA (ugd) |
| **Fragment1.F** | CGTAGGGATAACAGGGTAATGTCCAACAGACCCCGATAGACG | pKAPTURE capture cassette | BJ1 genomic DNA (galF) |
| **Fragment1.R** | CTTCGAGGAGTGCGTCA | pKAPTURE capture cassette | BJ1 genomic DNA (galF) |
| **Fragment2.F** | ACATTACCCTGTTATCCCTACGtcagatccttccgtatttagcc | pKAPTURE capture cassette | pSC101 (replicase and ori) |
| **Fragment3.R** | GGATAGGGATAACAGGGTAATgactcctgttgatagatccagtaatgac | pKAPTURE capture cassette | pSC101 (replicase and ori) |
| **Fragment3.F** | GTTTGGTAACGATTAAggaattttcactcccctcagaac | pKAPTURE capture cassette | Kanamycin resistance cassette |
| **Fragment3.R** | caggagtcATTACCCTGTTATCCCTAtcccgctcagaagaactcgt | pKAPTURE capture cassette | Kanamycin resistance cassette |
| **Fragment4.F** | CGTATTGTCATCGGTGAGCG | pKAPTURE capture cassette | BJ1 genomic DNA (ugd) |
| **Fragment4.R** | TTAATCGTTACCAAACAGATCGCGG | pKAPTURE capture cassette | BJ1 genomic DNA (ugd) |

**Supplementary Table S1 – Primers used in this study.**

| qseqid | sseqid | pident | length | mismatch | gapopen | qstart | qend | sstart | send | evalue | bitscore |
|---|---|---|---|---|---|---|---|---|---|---|---|
| TraN_Gamma_F (ompA) | **R1**_TraN | 98.339 | 602 | 10 | 0 | 1 | 602 | 1 | 602 | 0 | 1238 |
| TraN_Beta_pKpQIL (ompC) | **pKPN4_**TraN | 100 | 651 | 0 | 0 | 1 | 651 | 1 | 651 | 0 | 1347 |
| TraN_Beta_pKpQIL (ompC) | **p580**_TraN | 98.925 | 651 | 7 | 0 | 1 | 651 | 1 | 651 | 0 | 1335 |
| TraN_Alpha_pR100-1 (OmpW) | **p479**_TraN | 49.513 | 616 | 301 | 7 | 2 | 616 | 3 | 609 | 0 | 587 |
| OmpA_MG1655 | **BJ1**_OmpA | 85.955 | 356 | 40 | 2 | 1 | 346 | 1 | 356 | 0 | 589 |
| OmpA_MG1655 | **ST45**_OmpA | 85.955 | 356 | 40 | 2 | 1 | 346 | 1 | 356 | 0 | 589 |
| OmpA_MG1655 | **NTUH**_OmpA | 85.955 | 356 | 40 | 2 | 1 | 346 | 1 | 356 | 0 | 589 |

**Supplementary Table S2 – TraN annotation.** Results of a Blastp search from a database of TraN sequences against the plasmid sequences.

| Experiment_set | Donor | Recipient | Selection_donor | Selection_recipient | Selection_Transconjugant |
|---|---|---|---|---|---|
| E1 | DH10B | BJ1 | LB + Streptomycin | M63b1 + glucose 1% | M63b1 + glucose 1% + Kanamycin/Ertapenem |
| E1 | DH10B | NTUH | LB + Streptomycin | M63b1 + glucose 1% | M63b1 + glucose 1% + Kanamycin/Ertapenem |
| E1 | DH10B | ST45 | LB + Streptomycin | M63b1 + glucose 1% | M63b1 + glucose 1% + Kanamycin/Ertapenem |
| E2 | BJ1 | NTUH | LB + Chloramphenicol | M63b1 + Dulcitol 1% | M63b1 + Dulcitol 1% + Kanamycin/Ertapenem |
| E2 | BJ1 | ST45 | LB + Chloramphenicol | M63b1 + Dulcitol 1% | M63b1 + Dulcitol 1% + Kanamycin/Ertapenem |
| E2 | NTUH | BJ1 | M63b1 + Dulcitol 1% | LB + Chloramphenicol | LB + Chloramphenicol + Kanamycin/Ertapenem |

**Supplementary Table S3 – Selective plating strategy.**

# Supplementary Text S1

Scarless Serotype Swap protocol
In *Klebsiella pneumoniae*

## 1- Considerations

The aim of this protocol is to generate isogenic mutants encoding and expressing different capsule serotypes. The capsule locus of *Klebsiella pneumoniae (Kpn)* is defined as the minimal sequence leading to the expression of a functional and typeable capsule. The capsule locus of *Kpn* is typically defined as the genomic region located within the two core genes (i.e. shared between all *Kpn*) 5'-*galF* and *ugd-3'.* On the 3' side of *ugd* is located a *gmd/wbgU* gene (transcribed in the opposite direction), and the O-antigen locus, which typically starts with the *tagG* gene. Always keep in mind that any other gene present after *ugd* (especially if they are in the same transcription direction) could be involved in the capsule synthesis. This is referred to as the "the border dilemma".

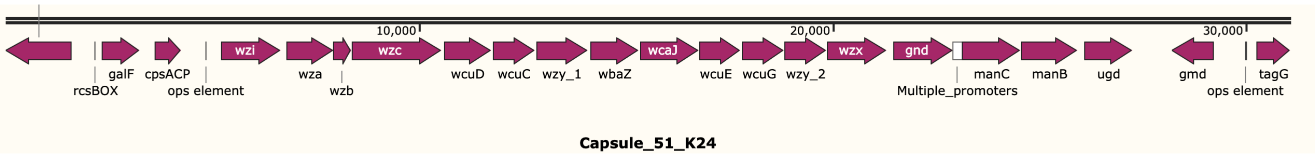


Figure 1 - Capsule locus of strain #51 of serotype K24. The capsule locus starts just before the rcsBOX (RcsAB binding site) and ends right after ugd. This typical organization is easy to exchange by homologous recombination: the borders are clearly *galF* and *ugd*.

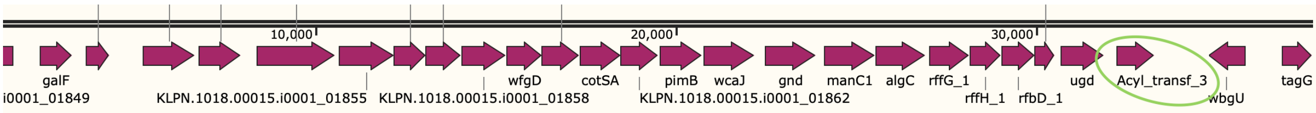


Figure 2 – The border dilemma of the K64 capsule. What is this Acyltransferase gene (green circle) and is it involved in capsule production and determining the K64 serotype?

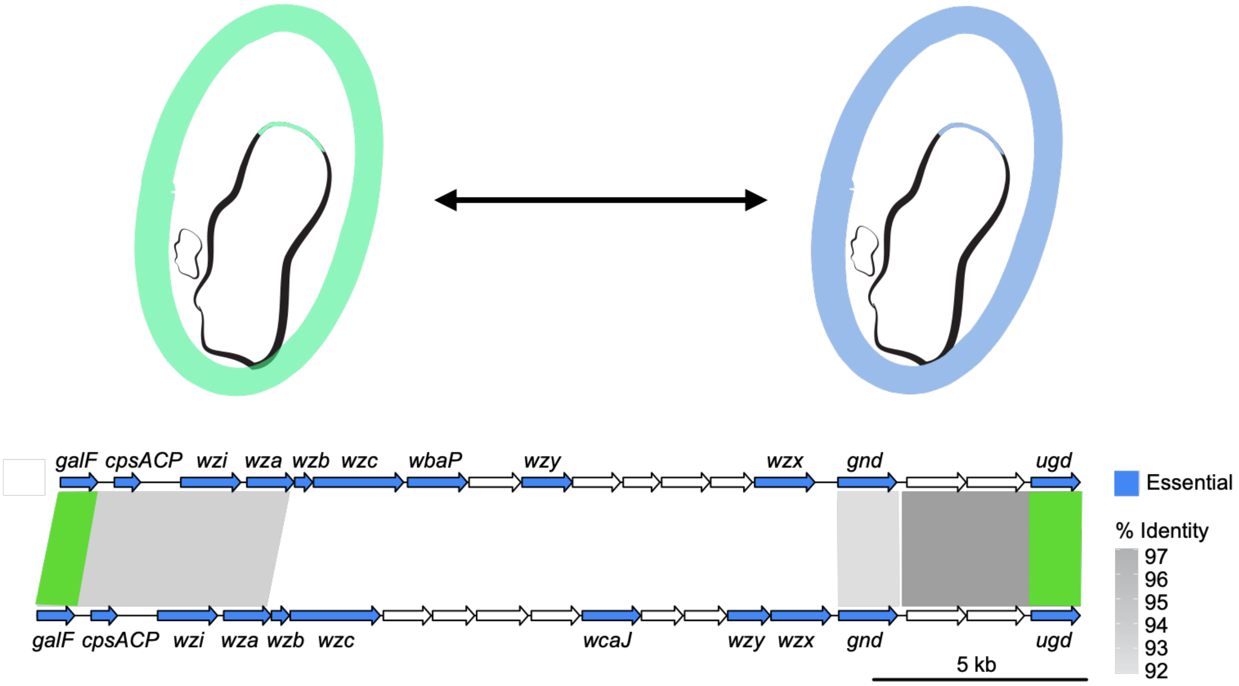


Figure 3 - Overview of a swap involving two strains. Capsules are thick colored edges. Capsule loci are colored chromosome segments. Two capsule loci are displayed on the bottom, with regions of high genetic similarity

(Recombination-prone). The two genes highlighted with green tracks will undergo the cross-overs leading to the swap.

## 2- Protocol

### A- Things needed

**Material needed:**

- Hot start Q5 polymerase (NEB)
- Electroporation cuvettes

**Strains needed:**

- Strain #751: source of capsule deletion cassette (KanR)
- Strain #745: source of pKAPTURE cassette (KanR)
- Strain #79: source of pKOBEG199 (TcR)
- Strain #672: source of pTKRED (SpecR, Thermosensitive must be cultured at 30deg)
- Strain #543: source of pMPIII (SpecR, Thermosensitive must be cultured at 30deg)

**NB:** It is very useful to generate large stocks of pKOBEG199/pTKRED/pMPIII. For pKOBEG199 and pTKRED, adding glucose to the culture can get higher yields and lower any counterselection from leaky expression of their enzymes.

**Primers needed:**

- Deletion cassette: #585/#590 – Expected Size 2.5kb – 68deg annealing, 1min30 elongation
- pKAPTURE cassette: #555/#560 – 4kb – 68deg annealing, 2min elongation
- Verification deletion: #88/#489 –3.5kb– 62deg annealing, 2min elongation
- Verification excision: #88/#489 –2kb– 62deg annealing, 2min elongation

### B- Protocol overview

In a serotype swap, the functionality of the cloned capsule locus is essential since it seems to be under high levels of purifying selection. An important control to test this, is to perform a complementation assay. Here is one way:

*If:*

- strain **A** encodes capsule locus KL**A** of serotype K**A** displays capsulated phenotype.
- strain ΔCapsule **A** encodes no capsule locus (genetic deletion) displays a non-capsulated phenotype.
- pKAPTURE_**A** encodes capsule locus KL**A** from strain **A**

*then:*

The introduction (and integration) of pKAPTURE_A in strain ΔCapsule **A** should lead to a capsulated phenotype. This is illustrated on Fig. 3. The **pK** strain (*trans* complemented) on Fig. 3 is the ideal stock strain to store a functional pKAPTURE since it is already in an adapted background and it is easy to pick capsulated colonies containing functional pKAPTURE vectors.

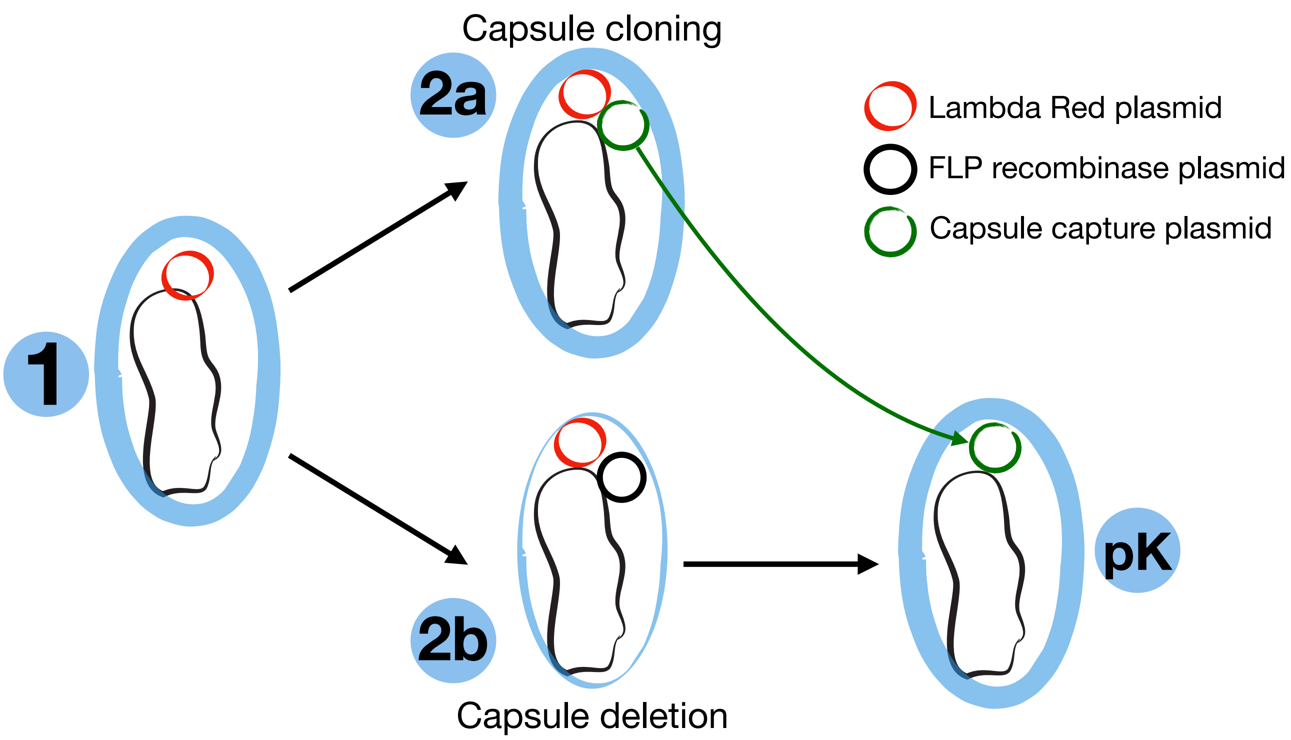


Figure 4 - Capsule complementation with pKAPTURE. Thick blue edges represent the capsule, thin blue edges represent the absence of capsule.

A serotype swap involving two strains can directly follow the *trans* complementation experiment. In this context, another strain with another capsule locus will be subject to capsule deletion and pKAPTURE integration. This is summarized on Fig. 4

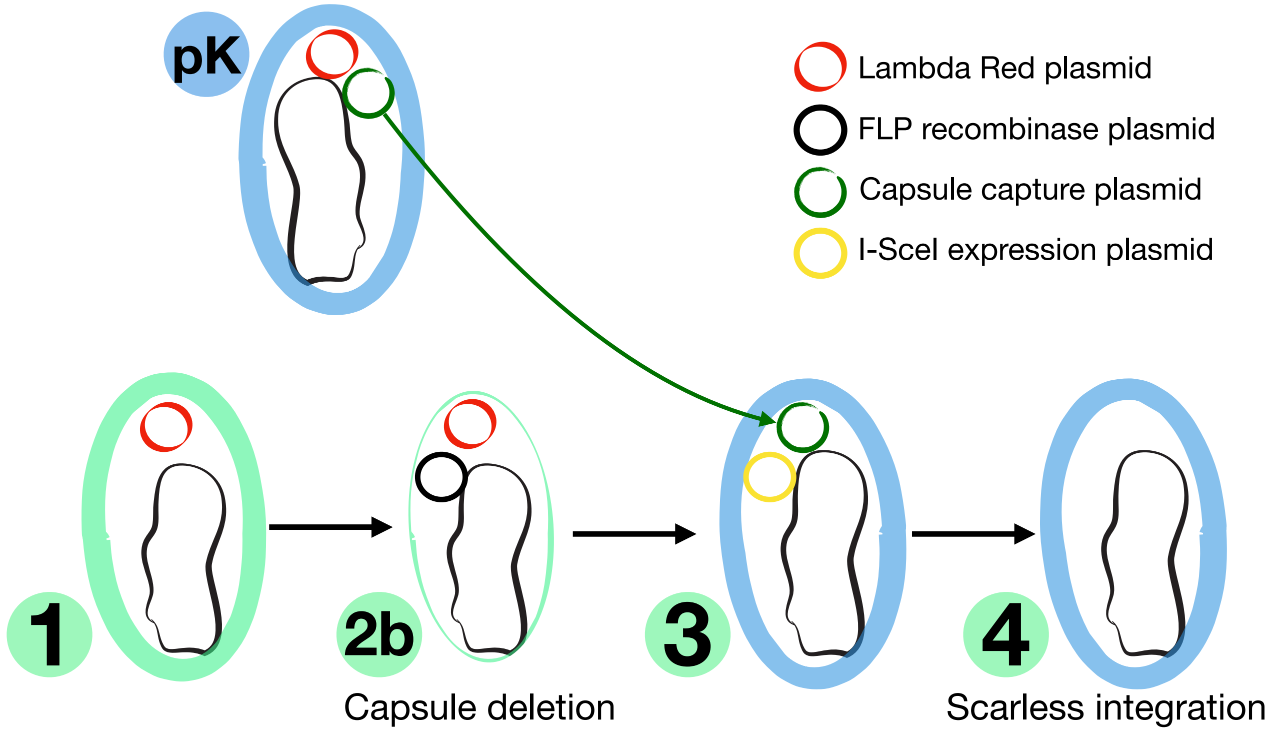


*Figure 5 - Overview of a serotype swap between a donor (blue) and recipient (originally green)*

## C- Generating capsule deletion mutants

1- Electroporate pKOBEG199 into your focus strain

*(See preparation of electrocompetent cells protocol)*
*(See also https://international.neb.com/protocols/2012/06/21/making-your-own-electrocompetent-cells )*

- It is not necessary to prepare the cells on ice.
- Add 0.2% Glucose to the recovery medium after electroporation
- pKOBEG199 harboring cells must be plated on LB-Tetracycline(15ug/mL)-Glucose(0.2%) plates to avoid leaky expression of Lambda Red enzymes

2- Electroporate the deletion cassette into your pKOBEG199-harboring strain

a. Prepare some deletion cassette by PCR (See Primers/Strains needed)
b. Run a gel to verify if the expected product was amplified
c. Use a PCR purification kit or drop-dialysis to remove salts from the PCR reaction
d. Prepare electrocompetent cells after inducing the lambda red enzymes present on pKOBEG199

1. Overnight your strain in LB+Tetracycline(15ug/mL)+Glucose(0.2%) (37°c)
2. Overday your strain in LB+Tetracycline(15ug/mL)+EDTA(7uM) until it reaches an OD of 0.5 (37°c)
3. At OD=0.5 add 0.2% L-Arabinose to induce Lambda Red **for 30min** (37°c)
4. Prepare the electrocompetent cells **on ice**
5. Electroporate 1uL of salt-free deletion cassette
6. Recover in 1mL LB at 37deg with shaking in culture tube for 1h
7. Plate 100uL of the outgrowth on LB+Kana (Add glucose if you are worried about leaky Lambda red expression)
8. Identify non-capsulated colonies, re-streak on LB+Kana to purify and and perform the verification of deletion PCR.

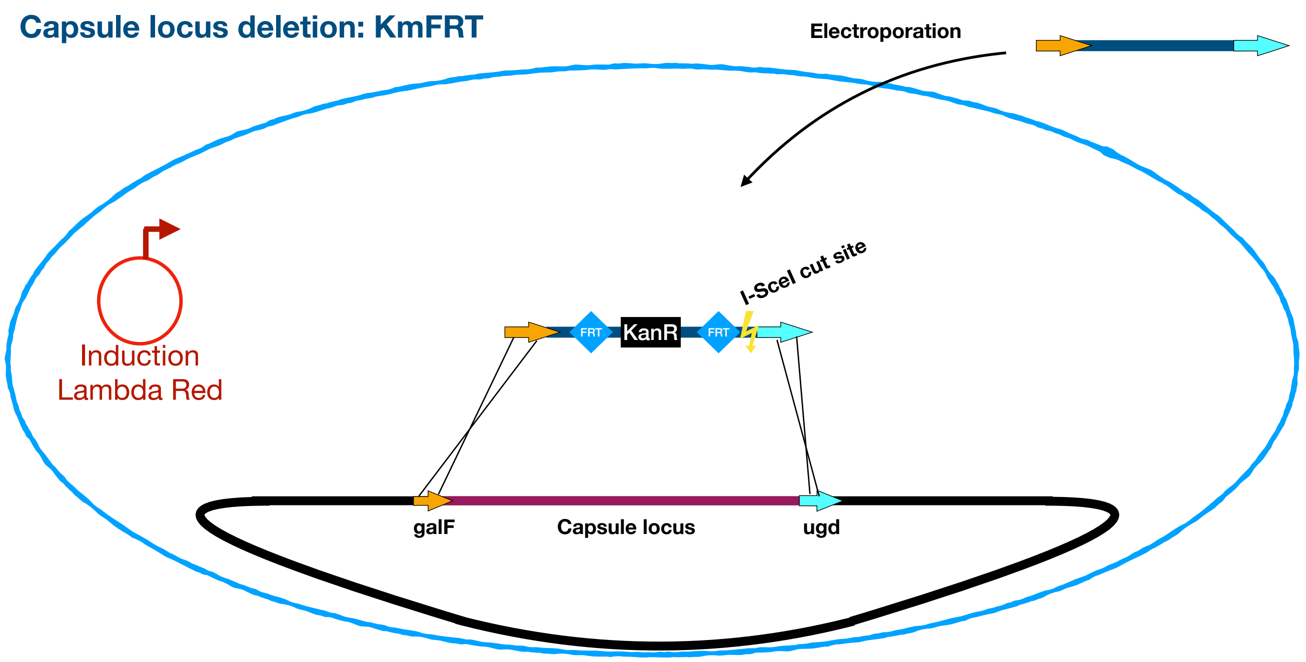


*Figure 6 - Deletion of the capsule locus*

3- Excise the KanMX resistance gene by expressing FLP:

1. Prepare electrocompetent cells of your non-capsulated cells (No need to do it on ice)

2. Electroporate 1uL of pMPIII miniprep
3. Recover in LB at 30°c for 1h30
4. Plate on LB+Spectinomycine (50ug/mL)
5. Incubate at 30°c
6. Pick 3 colonies and culture them overnight at 42°c to cure the pMPIII plasmid
7. Spread 100uL of $10^{-5}$ and $10^{-6}$ serial dilution and incubate at 37°c
8. Pick ~10 colonies and streak them in parallel on LB / LB+Kanamycin / LB+Tetracyclin / LB + Spectinomycin.
9. Identify a clone that only grows on LB and perform the verification of excision PCR. If it's good, this is your ΔCapsule mutant, congrats!

x

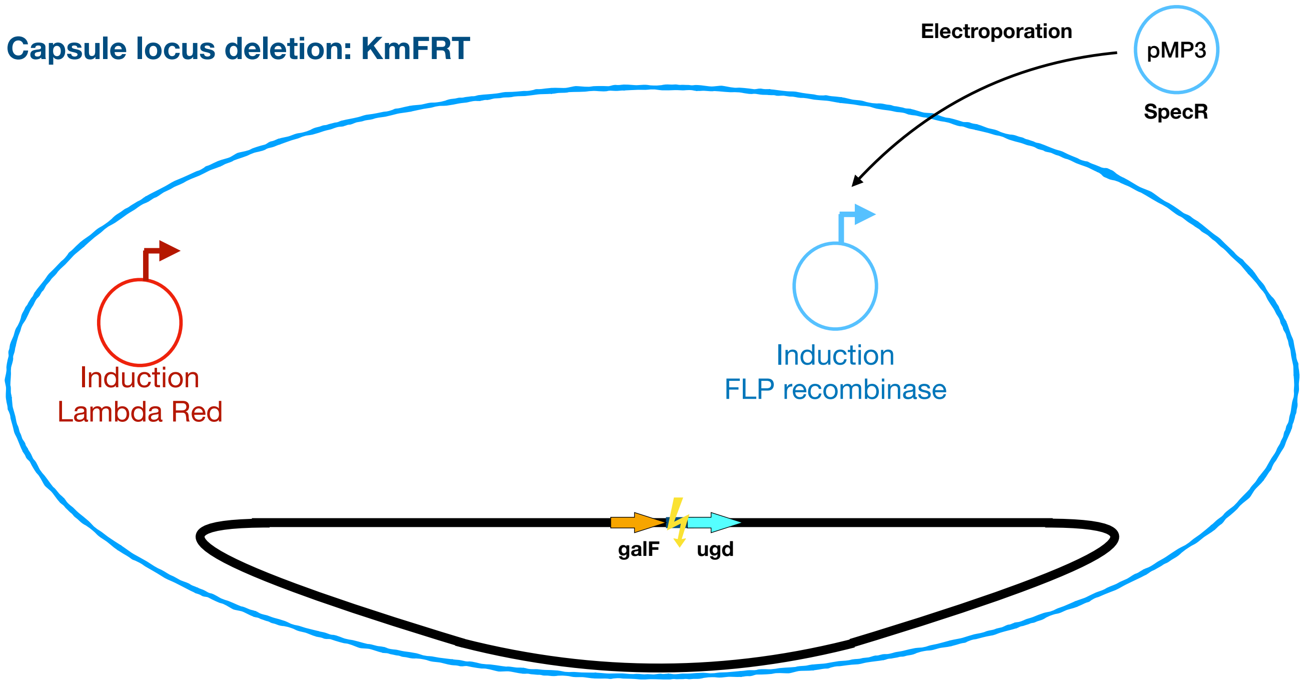


*Figure 7 - Excision of the kanMX-FRT marker*

## D- Generating pKAPTURE vectors

1- Electroporate pKOBEG199 into your strain carrying the serotype of interest. (See step A-1)

2- Electroporate the pKAPTURE into your pKOBEG199 strain:
   a. Prepare some pKAPTURE cassette by PCR (See Primers/Strains needed)
   b. Run a gel to verify if the expected product was amplified
   c. Use a PCR purification kit or drop-dialysis to remove salts from the PCR reaction
   d. Prepare electrocompetent cells after inducing the lambda red enzymes present on pKOBEG199:

1- Overnight your strain in LB+Tetracycline(15ug/mL)+Glucose(0.2%) (37°c)
2- Overday your strain in LB+Tetracycline(15ug/mL)+EDTA(7uM) until it reaches an OD of 0.2 (37°c)
3- At OD=0.2 add 0.2% L-Arabinose to induce Lambda Red **for 2 hours** (37°c)
4- Prepare the electrocompetent cells **on ice**
5- Electroporate 1uL of salt-free pKAPTURE cassette
6- Recover in 1mL LB at 37deg with shaking, in culture tube for 1h
7- Plate 100uL of the outgrowth on LB+Kana

8- Pick a few capsulated colonies, re-streak on LB+Kana and in parallel start independent cultures with each in LB+Kana+EDTA (7uM) overnight at 30°c to avoid capsule overproduction. Keep track of who is who.
9- Perform a miniprep for each culture. Elute in ddH2O and drop-dialysis all 30uL of the miniprep.
10- Electroporate this miniprep into your ΔCapsule mutant (Same strain as the pKATURE strain to do the complementation)
Add all 30uL of miniprep by using it to dilute the cells at the last step of electrocompetent cells preparation.
11- Recover in 1mL LB (Shaking, 37°c) and plate 100uL of the outgrowth.
12- Plate on LB+Kana.
13- Identify capsulated colonies and re-streak in parallel on LB / LB+Kana.
14- Isolate one colony that is: non-capsulated on LB and capsulated on LB+Kana. This is now your source of pKAPTURE, stock it. Well done!

## 2b Gap-Repair linear vector: pKAPTURE-lin

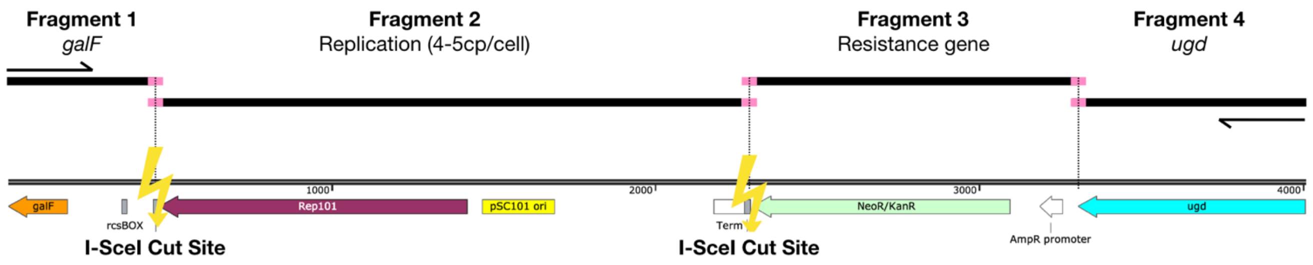


## 2b Gap-Repair linear vector: pKAPTURE-Kx

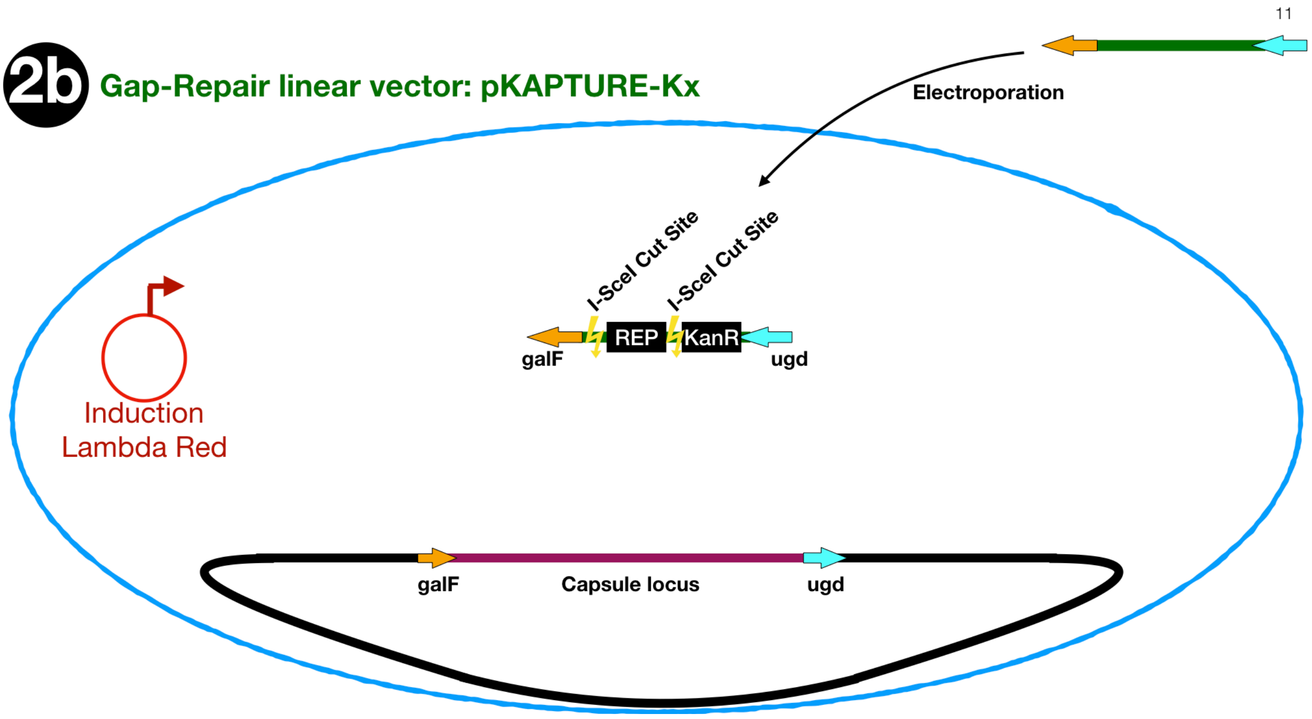

12

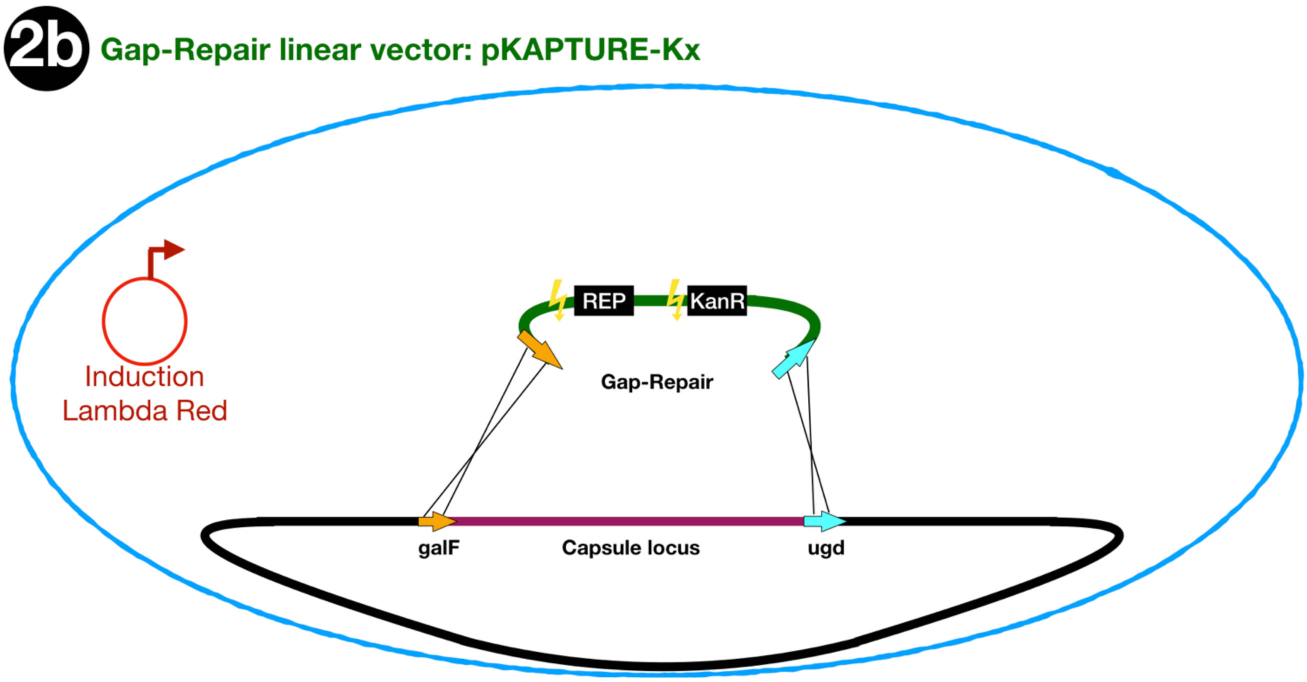
2b
Gap-Repair linear vector: pKAPTURE-Kx
REP
KanR
Gap-Repair
Induction
Lambda Red
galF
Capsule locus
ugd

13

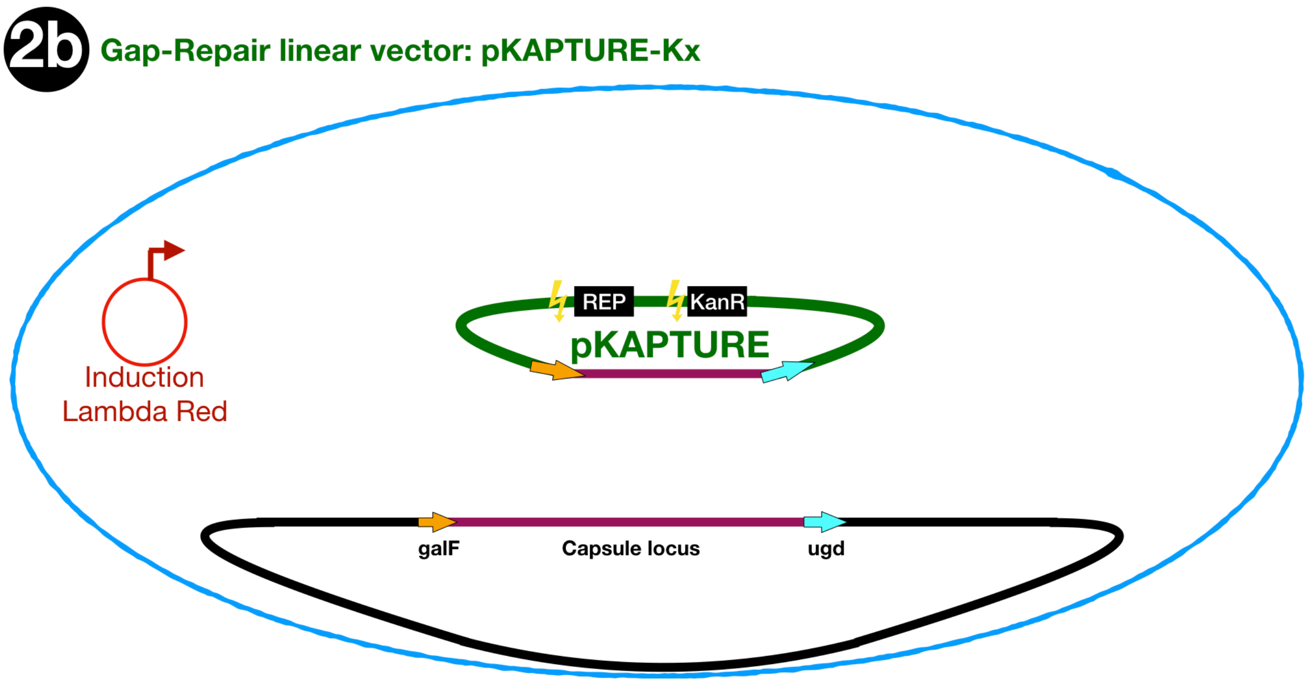
2b
Gap-Repair linear vector: pKAPTURE-Kx
REP
KanR
pKAPTURE
Induction
Lambda Red
galF
Capsule locus
ugd

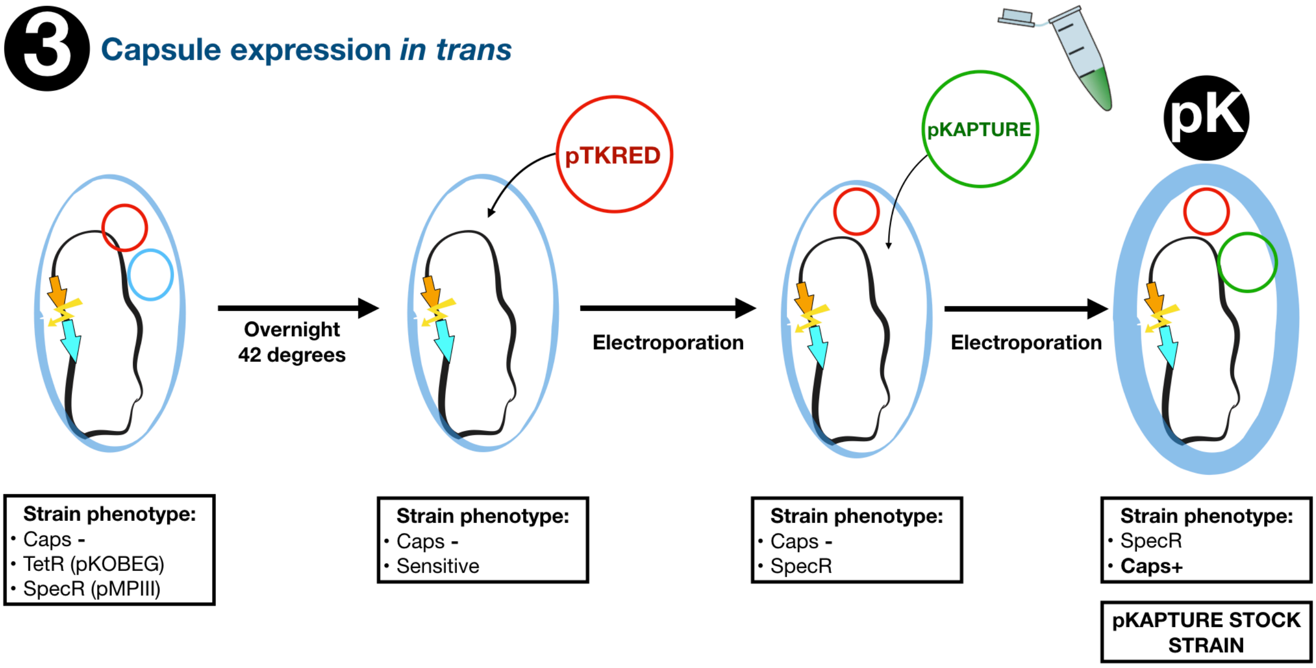


## E- Integrating capsule locus from pKAPTURE into Δcapsule mutants

1- Prepare a miniprep of your pKAPTURE from your stock strain (Step D.13)
   Elute in ddH2O and drop-dialysis the resulting 30uL
2- Prepare electrocompetent cells of your Δcapsule mutants (No need to do it on ice)
3- Electroporate 30uL of pKAPTURE miniprep by diluting the competent cells in it
4- Recover in 1mL LB at 37°c for about 1h (Shaking, culture tube)
5- Plate on LB+Kana and grow overnight at 37°c
6- Pick a capsulated colony, parallel streak on LB / LB+Kana
   (Must be non-caps on LB and capsulated on LB+Kana)
7- Inoculate a colony from LB+Kana into LB+Kana+EDTA (15mL) in the morning
8- Prepare electrocompetent cells once the culture reach OD~0.7 (no need to do it on ice)
9- Electroporate pTKRED
10- Recover at 30°c in 1mL LB+Kana+0.2%Glucose for 1h30 (Shaking, culture tube)
11- Plate on LB+Kana+Spectinomycine(50ug/mL)+Glucose(0.2%) and incubate at RT or 30°c
12- Pick several colonies and resuspend in 5mL M63b1 +Spectinomycine(100ug/mL) +0.2%L-Arabinose +0.2%Glyrerol
13- Cultivate at 30degrees (Shaking)
   You can plate at the end of the day and the next day on LB (+Glucose0.2%) at 42°c to cure pTKRED. (Adapt the dilution factor before plating according to the turbidity of the culture)
14- Identify capsulated colonies and parallel streak on LB / LB+Spec / LB+Kana
15- Identify a clone that only grows on LB
16- This is it, this is your swapped clone !
   Use primers specific to your new capsule locus to verify the strain

# Supplementary Text S2 - **Statistical analysis**

Variables

- **Strains**: BJ1 [26], ST45 [51] and NTUH [56]
- **MPF**: Mating-pair formation type
- **Plasmid**: plasmid name
- **Recipient_Genotype**: capsule genotype either non-capsulated (Delta) or various serotypes (K1, K2, K3, K24) of the recipient in conjugation pairs
- **Donor_Genotype**: capsule genotype either non-capsulated (Delta) or various serotypes (K1, K2, K3, K24) of the donor in conjugation pairs
- **CE:** Log-transformed conjugation efficiency
- **Volume**: effective volume measured by colony dissolution
- **Norm_production**: Chemical quantification of capsule amount by uronic acid dosage

**Statistics 1**. To test the hypothesis that MPF differ in conjugation efficiencies we used a linear mixed model where the MPF type was the fixed effect, and the plasmid identity was the random effect. The response variable was the conjugation efficiency (log transformed).

**Fixed effects parameter estimates**

| Term | Estimate | Std Error | t Ratio | Prob>\|t\| |
|---|---|---|---|---|
| Intercept | -9.739662 | 0.1480876 | -65.77 | <0.0001 |
| MPF[F] | -0.647401 | 0.2276429 | -2.84 | 0.0316 |
| MPF[I] | 0.7955165 | 0.2047639 | 3.89 | 0.0091 |

The test of the fixed effects (MPF) was significant (Fratio= 7.752446, P=0.0233, F test).
Table 1 – Fixed effects parameter estimates for Statistics 1

**Random Effects Covariance Parameter Estimates**

| Variance component | Estimate | Std Error | 95% Lower | 95% Upper |
|---|---|---|---|---|
| Plasmid | 0.1580173 | 0.1076183 | -0.052911 | 0.3689453 |
| Residual | 0.9491 | 0.0733431 | 0,8202874 | 1.1110022 |

Table 2 – Random Effects Covariance Parameter Estimates for Statistics 1

**Statistics 2**. Comparisons of all pairs of donor/recipient genotypes using non-parametric tests and HSD analysis.

**Dataset E1**. The Wilcoxon test showed a significant difference across the MPF types (Chi2 approximation=85.1846, DF=2, P<0.001).

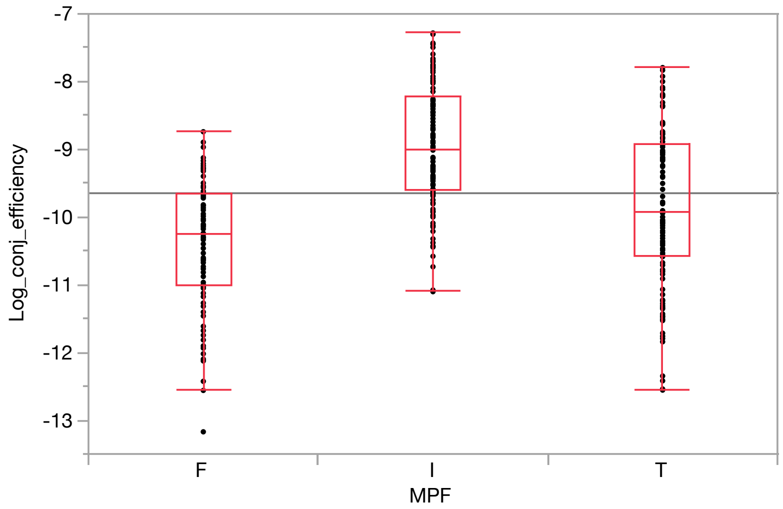


Figure 1 – Conjugation efficiency (log) between mating pair formation types (MPF) for Dataset E1
Log_cong_efficiency: log-transformed conjugation efficiency.

HSD Threshold matrix (Positive values show pairs of means significantly different at alpha=0.05).

| | I | T | F |
|---|---|---|---|
| I | -0,2987 | 0,6064 | 1,1048 |
| T | 0,6064 | -0,3046 | 0,1941 |
| F | 1,1048 | 0,1941 | -0,3610 |

In dataset E1 (conjugation experiments from *E.coli* to *K .pneumoniae*), we can assess the interaction with an ANOVA since there are several independent plasmids with different MPF. However, the interaction term between MPF and recipient genotype is non-significant.

| | Df | Sum Sq | Mean Sq | F-value | p-value | |
|---|---|---|---|---|---|---|
| Recipient | 2 | 117.16 | 58.58 | 111.832 | <2,00E-16 | *** |
| Recipient Genotype | 3 | 12.30 | 4.10 | 7.828 | 4.62e-05 | *** |
| MPF | 2 | 121.27 | 60.63 | 115.755 | <2,00E-16 | *** |
| Recipient Genotype:MPF | 6 | 4.34 | 0.72 | 1.381 | **0.221** | |
| Residuals | 330 | 172.85 | 0.52 | | | |

Table 3 – ANOVA table for Statistics 2

**Dataset E2**. The Wilcoxon test showed a significant difference across the MPF types (Chi2 approximation= 238.4514, DF=2, P<0.001).

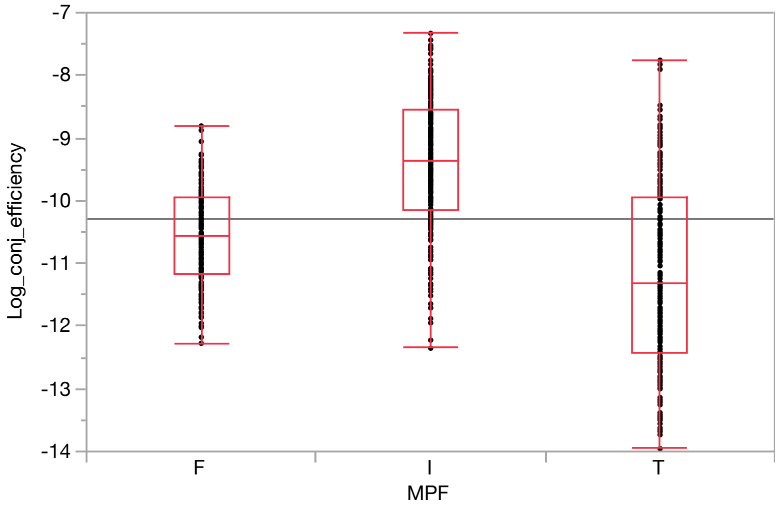


Figure 2 – Conjugation efficiency (log) between mating pair formation types (MPF) for dataset E2.
Log_cong_efficiency: log-transformed conjugation efficiency.

HSD Threshold matrix (Positive values show pairs of means significantly different at alpha=0.05).

| | I | F | T |
|---|---|---|---|
| I | -0,2168 | 0,9588 | 1,5858 |
| F | 0,9588 | -0,2504 | 0,3764 |
| T | 1,5858 | 0,3764 | -0,2429 |

Interaction with capsule type:

**Statistics 3**. We made a linear mixed model where we put together as fixed effects the MPF type of the plasmid and the serotypes of the donor and recipient, while conditioning (random effects) for the identity of the donor and recipient chassis strains. The response variable was the efficiency of conjugation (log transformed). This analysis was done with dataset E2 (conjugation to and from K. pneumoniae), since in E1 there is no variation in the donor. The AICc was 1464,0046 and the BIC 1528,3838.

**Fixed effects parameter estimates**

| Term | Estimate | Std Error | t Ratio | Prob>\|t\| |
|---|---|---|---|---|
| Intercept | -10,16391 | 0,8097412 | -12.55 | 0.0537 |
| MPF[F] | -0,200576 | 0,0326421 | -6.14 | <0.0001 |
| MPF[I] | 0,9923876 | 0,0304167 | 32.63 | <0.0001 |
| Donor_Genotype[Delta] | 0,6610264 | 0,0443087 | 14.92 | <0.0001 |
| Donor_Genotype[K1] | -0,517907 | 0,0451052 | -11.48 | <0.0001 |
| Donor_Genotype[K2] | -0,122701 | 0,0444123 | -2.76 | 0.0059 |
| Donor_Genotype[K3] | 0,0896048 | 0,0444134 | 2.02 | 0.0440 |
| Recipient_Genotype[Delta] | 0,4106946 | 0,0443087 | 9.27 | <0.0001 |
| Recipient_Genotype[K1] | -0,365228 | 0,0448612 | -8.14 | <0.0001 |
| Recipient_Genotype[K2] | -0,219808 | 0,0446298 | -4.93 | <0.0001 |
| Recipient_Genotype[K3] | 0,157139 | 0,0445191 | 3.53 | <0.0001 |

Table 4 – Fixed effects parameter estimates for Statistics 3

**Fixed effects tests**

| Term | DF | F ratio | P |
|---|---|---|---|
| MPF | 2 | 591.83095 | <0.0001 |
| Donor_Genotype | 4 | 74.374979 | <0.0001 |
| Recipient_Genotype | 4 | 37.794612 | <0.0001 |

Table 5 – Fixed effects tests for Statistics 3

**Statistics 4.** we used standard least squares to model the conjugation efficiency (Log transformed) in function of the MPF type and the serotypes of donor and recipients. Here, we used only the data of conjugation between *K. pneumoniae* strains (dataset E2, since for dataset E1 the donor is never capsulated). This resulted in a significant linear model ($R^2$=0.43, p<0.001, F test), where the tests on the three variables revealed significant effect (p<0.001), but the interaction term was non-significant (p>0.9, same test). Dataset E2.

**Analysis of variance.** The test rejected the null hypothesis of no association (F ratio= 21.6925, P<0.0001, F test).

| Source | DF | SS | Mean square |
|---|---|---|---|
| Model | 26 | 622.4406 | 23.94 |
| Error | 737 | 813.3594 | 1.1036 |
| Total | 763 | 1435.8 | |

Table 6 – Analysis of variance table for Statistics 4

**Effect tests.** The individual terms have a significant effect, but their interaction does not.

| Source | DF | SS | F Ratio | P |
|---|---|---|---|---|
| Donor_Genotype | 4 | 105.828 | 23.9732 | <0.0001 |
| Recipient_Genotype | 4 | 53.34829 | 12.0850 | <0.0001 |
| Donor_Genotype*Recipient_Genotype | 16 | 2.18865 | 0.1239 | 1.0 |
| MPF | 2 | 472.56652 | 214.1006 | <0.0001 |

Table 6 – Effect tests for Statistics 4

**Statistics 5**. Individual ANOVAs where each of the variables is fitted to the conjugation efficiency (Log transformed). Dataset E2.

| Variable | $R^2$ | Observations | SS | DF | P |
|---|---|---|---|---|---|
| MPF | 0.322163 | 764 | 462.5616 | 2 | <0.0001 |
| Donor_Genotype | 0.068203 | 764 | 97.9258 | 4 | <0.0001 |
| Recipient_Genotype | 0.033681 | 764 | 48.3595 | 4 | <0.0001 |

Table 7 – Individual ANOVAs summary table for Statistics 5

**Statistics 6a**. We fitted a linear mixed model with the **cell volume** as a fixed effect and the chassis strain as a random effect and the conjugation efficiency as the response variable (Log transformed). The AICc was 9.7660305 and the BIC 8.5982313.

**Fixed effects parameter estimates**

| Term | Estimate | Std Error | t Ratio | Prob>\|t\| |
|---|---|---|---|---|
| Intercept | -9.563545 | 0.5998599 | -15.94 | 0.0033 |
| Volume | -0.017874 | 0.0026548 | -6.73 | <0.0001 |

Table 8 – Fixed effects parameter estimates for Statistics 6a

The test of the fixed effects (Volume) was significant (Fratio= 45.330323, P<0.0001, F test).

**Random Effects Covariance Parameter Estimates**

| Variance component | Estimate | Std Error | 95% Lower | 95% Upper |
|---|---|---|---|---|
| Strain | 1,0530009 | 1,0568503 | -1,018388 | 3,1243894 |
| Residual | 0,0186297 | 0,0079439 | 0,0093487 | 0,0537067 |

Table 9 – Random Effects Covariance Parameter Estimates for Statistics 6a

**Statistics 6b**. We fitted a linear mixed model with the **capsule quantification** (norm_production) as a fixed effect and the chassis strain as a random effect. The conjugation efficiency as the response variable (Log transformed). The $R^2$ of 0.976832.

**Fixed effects parameter estimates**

| **Term** | **Estimate** | **Std Error** | **t Ratio** | **Prob>\|t\|** |
|---|---|---|---|---|
| Intercept | -9.518452 | 0.652195 | -14.59 | 0.0038 |
| Mean(norm_production) | -0.303604 | 0.04975 | -6.10 | <0.0001 |

Table 10 – Fixed effects parameter estimates for Statistics 6b

The test of the fixed effects (capsule production) was significant (Fratio=37.2421, P<0.0001, F test).

**Random Effects**

| **Term** | **Estimate** | **Std Error** | **t Ratio** | **Prob>\|t\|** |
|---|---|---|---|---|
| Strain[26] | 1.1285075 | 0.645665 | 1.75 | 0.2215 |
| Strain[51] | -1.092931 | 0.645421 | -1.69 | 0.2316 |
| Strain[56] | -0.035577 | 0.645068 | -0.06 | 0.9610 |

Table 11 – Random effects parameter estimates for Statistics 6b

**Regression Plot**

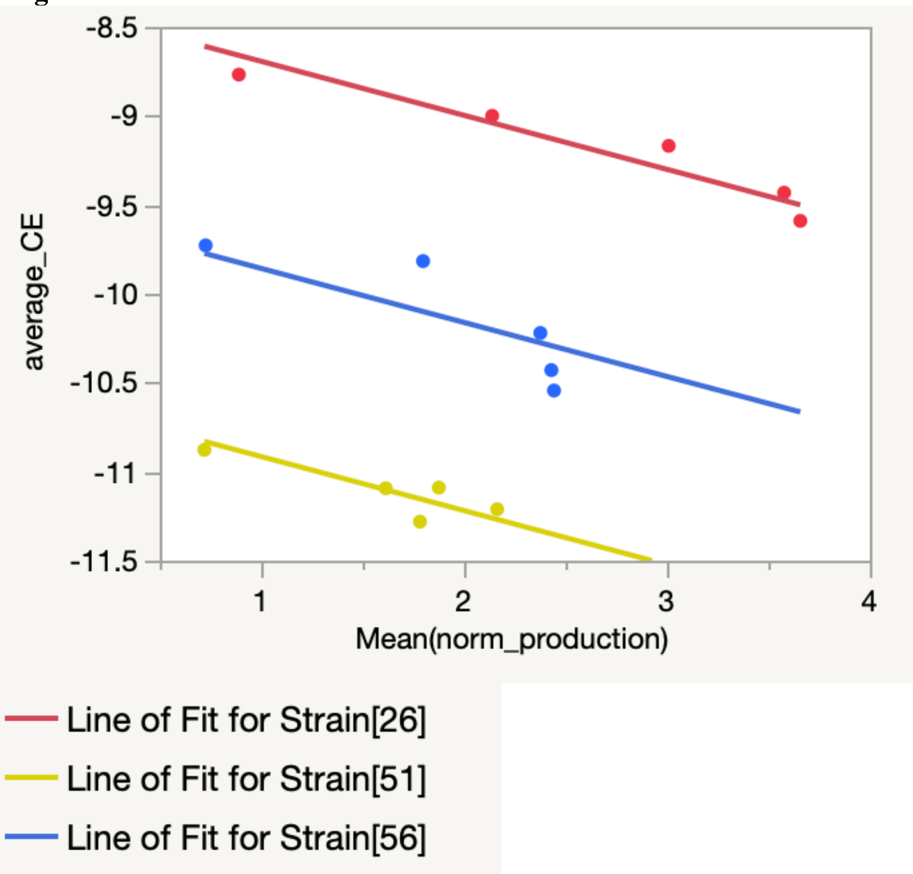


Figure 3 - Mean of log-transformed conjugation efficiencies (Average_CE) vs. mean of capsule quantity by chemical dosage (Mean(norm_production)). Lines corresponds to individual regression lines for each strain.
Average_CE: Mean of log-transformed conjugation efficiencies.
Mean(norm_production): Mean of capsule quantity by chemical dosage

**Statistics 6c.Same statistics with Strain and Volume as a fixed effect (standard least squares).**

The fit is very similar to the one of the mixed models. The $R^2$ is 0.980021.

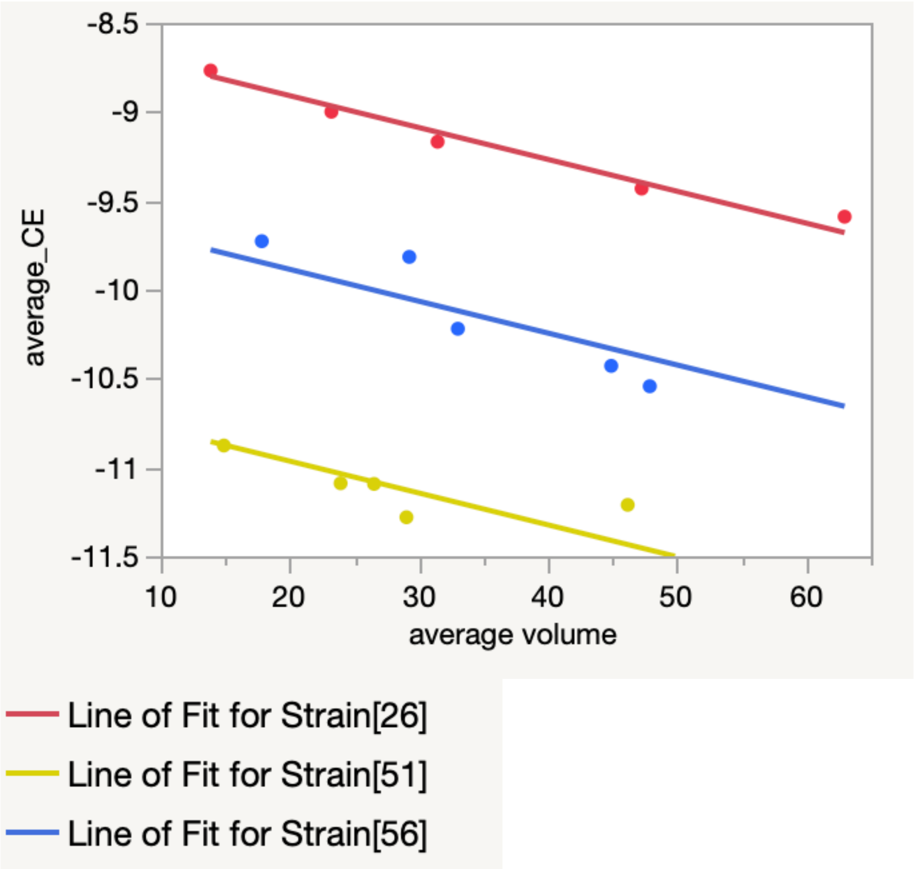


Figure 4 - Mean of log-transformed conjugation efficiencies (Average_CE) vs. mean of effective volume (Average volume). Lines corresponds to individual regression lines for each strain.
Average_CE: Mean of log-transformed conjugation efficiencies.
Average volume : Mean of effective volume

**Analysis of Variance**

| Source | DF | Sum of Squares | Mean Square | F Ratio | Prob > F |
|---|---|---|---|---|---|
| Model | 3 | 10.051793 | 3.35060 | 179.8579 | <.0001* |
| Error | 11 | 0.204921 | 0.01863 | | |
| C. Total | 14 | 10.256713 | | | |

**Parameter Estimates**

| Term | Estimate | Std Error | t Ratio | Prob>\|t\| |
|---|---|---|---|---|
| Intercept | -9.561808 | 0.093962 | -101.8 | <.0001* |
| Strain[26] | 1.0107618 | 0.050445 | 20.04 | <.0001* |
| Strain[51] | -1.04472 | 0.051377 | -20.33 | <.0001* |
| average volume | -0.017927 | 0.002654 | -6.75 | <.0001* |

**Effect Tests**

| Source | DF | Sum of Squares | F Ratio | P |
|---|---|---|---|---|
| Strain | 2 | 10.010857 | 268.6881 | <0.0001 |
| average volume | 1 | 0.849761 | 45.6146 | <0.0001 |

Table 12 – Analysis of variance, parameter estimates and effect tests for Statistics 6c.

**Statistics 6d.Same statistics with Strain and Amount of capsule as a fixed effect (standard least squares)**. The fit is very similar to the one of the mixed models. The $R^2$ is 0.976851,

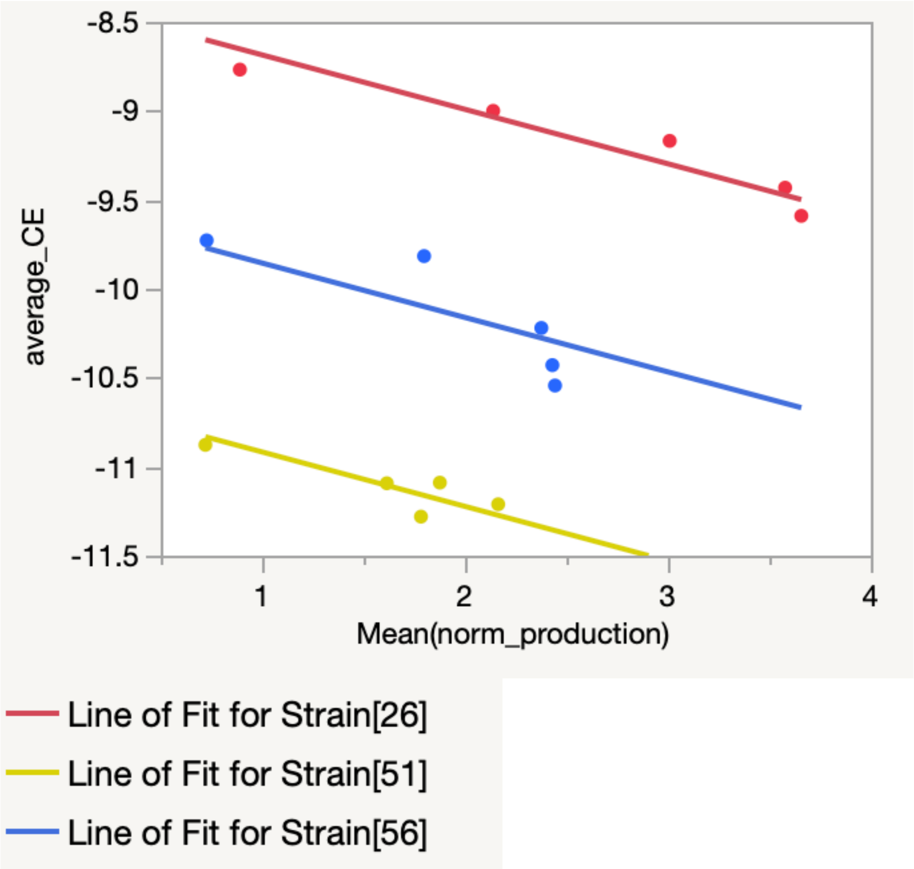


Figure 5 - Mean of log-transformed conjugation efficiencies (Average_CE) vs. mean of capsule quantity by chemical dosage (Mean(norm_production)). Lines corresponds to individual regression lines for each strain.

**Analysis of Variance**

| Source | DF | Sum of Squares | Mean Square | F Ratio | P |
|---|---|---|---|---|---|
| Model | 3 | 10.019283 | 3.33976 | 154.7293 | <0.0001 |
| Error | 11 | 0.237430 | 0.02158 | | |
| C. Total | 14 | 10.256713 | | | |

**Parameter Estimates**

| Term | Estimate | Std Error | t Ratio | P |
|---|---|---|---|---|
| Intercept | -9.51371 | 0.110159 | -86.36 | <0.0001 |
| Strain[26] | 1.1337466 | 0.060769 | 18.66 | <0.0001 |
| Strain[51] | -1.09776 | 0.058103 | -18.89 | <0.0001 |
| Mean(norm_production) | -0.305883 | 0.049711 | -6.15 | <0.0001 |

**Effect Tests**

| Source | DF | Sum of Squares | F Ratio | Prob > F |
|---|---|---|---|---|
| Strain | 2 | 9.5843772 | 222.0194 | <0.0001 |
| Mean(norm_production) | 1 | 0.8172519 | 37.8628 | <0.0001 |

Table 13 – Analysis of variance, parameter estimates and effect tests for Statistics 6d.

**Statistics 6e**. We fitted a linear mixed model with the capsule quantification as a fixed effect and the chassis strain as a fixed effect. The average volume as the response variable. R2= 0.795468

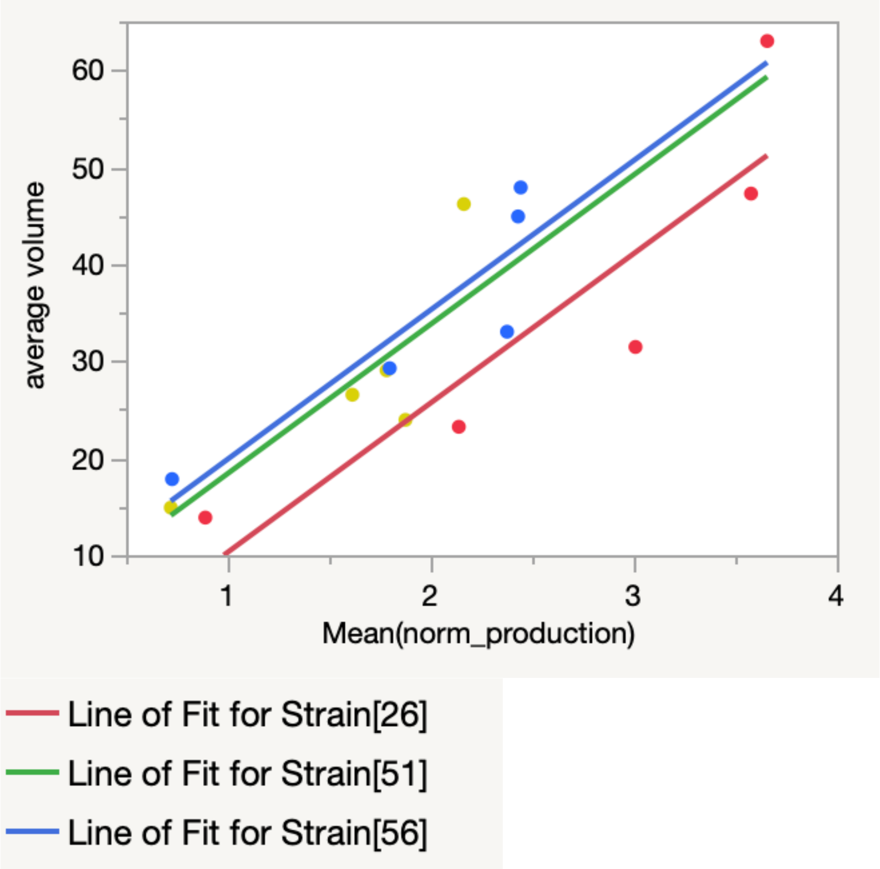


Figure 6 - Mean of effective volume (Average volume). vs. mean of capsule quantity by chemical dosage (Mean(norm_production)). Lines corresponds to individual regression lines for each strain.
R2= 0.795468

**Analysis of Variance**

| **Source** | **DF** | **Sum of Squares** | **Mean Square** | **F Ratio** |
|---|---|---|---|---|
| Model | 3 | 2237.8096 | 745.937 | 14.2605 |
| Error | 11 | 575.3885 | 52.308 | **Prob > F** |
| C. Total | 14 | 2813.1981 | | 0.0004* |

**Parameter Estimates**

| **Term** | **Estimate** | **Std Error** | **t Ratio** | **Prob>\|t\|** |
|---|---|---|---|---|
| Intercept | 0.7978185 | 5.422918 | 0.15 | 0.8857 |
| Mean(norm_production) | 15.389313 | 2.447156 | 6.29 | <.0001* |
| Strain[26] | -5.899412 | 2.991534 | -1.97 | 0.0743 |
| Strain[51] | 2.2076116 | 2.860275 | 0.77 | 0.4565 |

**Effect Tests**

| **Source** | **DF** | **Sum of Squares** | **F Ratio** | **Prob > F** |
|---|---|---|---|---|
| Mean(norm_production) | 1 | 2068.6344 | 39.5472 | <.0001* |
| Strain | 2 | 215.4365 | 2.0593 | 0.1739 |

Table 14 – Analysis of variance, parameter estimates and effect tests for Statistics 6e.

**Statistics 7**. Linear mixed model with the cell volume as a fixed effect, the chassis strain **and the serotype** as a random effect ($R^2$=0.98).

**Fixed effects parameter estimates**

| Term | Estimate | Std Error | t Ratio | Prob>\|t\| |
|---|---|---|---|---|
| Intercept | -9.572305 | 0.602318 | -15.89 | 0.0032 |
| Volume | -0.01777 | 0.003149 | -5.64 | 0.0055 |

Table 14 – Fixed effects parameter estimates for Statistics 7.
The test of the fixed effects (Volume) was significant (Fratio= 31.84, P=0.005, F test).

**Statistics 8**.

## Analysis of Variance

Response: # of defence systems
Serotype: K1, K2, K3, K24

| | Df | Sum Sq | Mean Sq | F value | Pr(>F) |
|---|---|---|---|---|---|
| Serotype | 3 | 73.13 | 24.378 | 1.4743 | 0.2256 |
| Residuals | 108 | 1785.79 | 16.535 | | |

Table 15 – Analysis of variance table for Statistics 8.

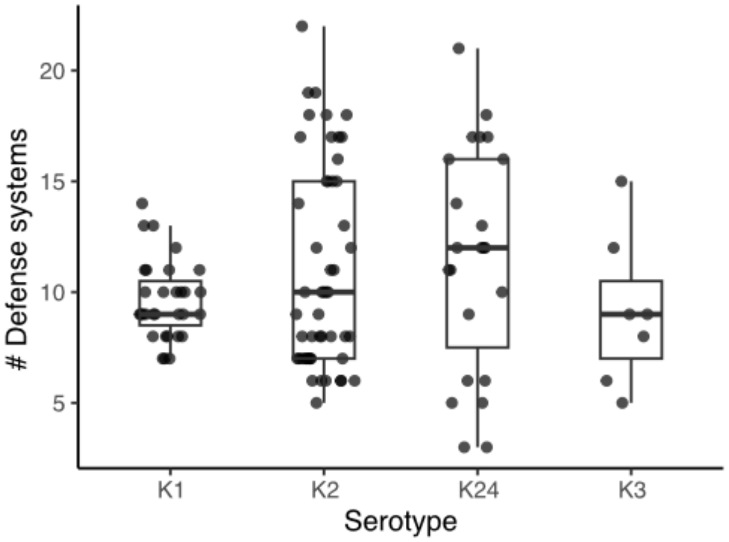


Figure 7 – Number of defense systems identified by DefenseFinder in genomes of different serotypes.

**Statistics 9**.

We put together K1 and K2 as strains with **large** capsules (associated with hyper-virulence), and K24 and K3 with **small** capsules (non-associated with hyper-virulence). We fit a Generalized Additive Model with the number of plasmid acquired in terminal branch as response, the type of plasmid (MPF F, T, I or Others) and strain serotype as fixed effect, and the log-transformed branch length as a smoothed function. The intercepts are Serotype_Large and Type_Others.

Family: Poisson
Link function: log

**Parametric coefficients:**

| | Estimate | Std. Error | z-value | Pr(>\|z\|) | |
|---|---|---|---|---|---|
| (Intercept) | -0.3186 | 0.1347 | -2.365 | 0.0180 | * |
| Serotype_Small | 0.4313 | 0.1745 | 2.472 | 0.0134 | * |
| TypeT | -1.7540 | 0.2553 | -6.871 | 6.38e-12 | *** |
| TypeI | -2.8526 | 0.4199 | -6.794 | 1.09e-11 | *** |
| TypeF | -1.6487 | 0.2442 | -6.752 | 1.46e-11 | *** |

**Approximate significance of smooth terms:**

| | edf | Ref.df | Chi.sq | p-value | |
|---|---|---|---|---|---|
| s(log_branch_length) | 3.253 | 4.109 | 47.12 | <2e-16 | *** |

R-sq.(adj) = 0.354 ; Deviance explained = 39.7%
UBRE = -0.19739 ; Scale est. = 1; n = 408

Table 16 – Parametric coefficients and significance of smooth terms table for Statistics 9.
