# Supplementary material for: Capsules and their traits shape phage susceptibility and plasmid conjugation efficiency
Source: Nat Commun. 2024 Mar 6;15:2032. doi: 10.1038/s41467-024-46147-5 (PMC10918111; doi:10.1038/s41467-024-46147-5)
Supplement: Supplementary file 3 — Description of Additional Supplementary Files [file 41467_2024_46147_MOESM3_ESM.pdf]

Description of Additional Supplementary Files

Supplementary Data 1: Strains.xlsx – List of strains used in this study.

Supplementary Data 2: Plasmids.xlsx – List of plasmids used in this study.

Supplementary Data 3: Deletion_cassette.xlsx – Sequence of the deletion cassette used to delete the capsule loci.

Supplementary Data 4: Capture_cassette.xlsx – Sequence of the genomic capture cassette used to clone capsule loci.

Supplementary Data 5: Plasmids_sequences.xlsx – Assemblies and annotations of plasmids used in this study.

Supplementary Data 6: TraN_OmpA_alleles.xlsx – TraN and OmpA reference alleles used in this study.

Supplementary Data 7: Breseq.xlsx – Breseq outputs for the mutants constructed in this study.

Supplementary Data 8: Genomic_dataset.xlsx – List of NCBI RefSeq genomes used in this study.

Supplementary Data 9: Pangenome_table.xlsx – Pangenome table for the NCBI Refseq genomes used in this study.

Supplementary Data 10: Phylogenetic_tree.xlsx – Phylogenetic tree of the NCBI Refseq genomes used in this study.

Supplementary Data 11: Capsule_regulators.xlsx – List of capsule regulators identified in the genomes of the strains used in this study.

Supplementary Data 12: RefSeq_Plasmids.xlsx – List of NCBI RefSeq Plasmids used in this study.
