# Supplementary material for: Capsules and their traits shape phage susceptibility and plasmid conjugation efficiency
Source: Nat Commun. 2024 Mar 6;15:2032. doi: 10.1038/s41467-024-46147-5 (PMC10918111; doi:10.1038/s41467-024-46147-5)
Supplement: Supplementary file 16 — Reporting Summary [file 41467_2024_46147_MOESM16_ESM.pdf]

nature portfolio

Corresponding author(s): Matthieu Haudiquet

Last updated by author(s): Dec 6, 2023

Reporting Summary

Nature Portfolio wishes to improve the reproducibility of the work that we publish. This form provides structure for consistency and transparency in reporting. For further information on Nature Portfolio policies, see our Editorial Policies and the Editorial Policy Checklist.

Statistics

For all statistical analyses, confirm that the following items are present in the figure legend, table legend, main text, or Methods section.

n/a	Confirmed	
☐	☒	The exact sample size (*n*) for each experimental group/condition, given as a discrete number and unit of measurement
☐	☒	A statement on whether measurements were taken from distinct samples or whether the same sample was measured repeatedly
☐	☒	The statistical test(s) used AND whether they are one- or two-sided<br>*Only common tests should be described solely by name; describe more complex techniques in the Methods section.*
☐	☒	A description of all covariates tested
☐	☒	A description of any assumptions or corrections, such as tests of normality and adjustment for multiple comparisons
☐	☒	A full description of the statistical parameters including central tendency (e.g. means) or other basic estimates (e.g. regression coefficient) AND variation (e.g. standard deviation) or associated estimates of uncertainty (e.g. confidence intervals)
☐	☒	For null hypothesis testing, the test statistic (e.g. *F*, *t*, *r*) with confidence intervals, effect sizes, degrees of freedom and *P* value noted<br>*Give P values as exact values whenever suitable.*
☒	☐	For Bayesian analysis, information on the choice of priors and Markov chain Monte Carlo settings
☐	☒	For hierarchical and complex designs, identification of the appropriate level for tests and full reporting of outcomes
☐	☒	Estimates of effect sizes (e.g. Cohen's *d*, Pearson's *r*), indicating how they were calculated

Our web collection on statistics for biologists contains articles on many of the points above.

Software and code

Policy information about availability of computer code

Data collection

We retrieved all the K. pneumoniae complete genomes available in the NCBI non-redundant RefSeq database, accessed in March 2021, along with their gene annotations. This resulted in a set of 730 genomes containing 2386 associated plasmids. The pairwise genetic distances between all genomes of the species was calculated using MASH [132]. Strains that were too divergent (MASH distance >6%) to the reference strain or too similar (<0.0001) to any other strains were removed from further analysis. A total of 623 genomes were analysed. To identify defence systems, we ran DefenseFinder v1.0.9 with default options [65]. The information on the genomes (including accession numbers) is available in Supplementary Table S6.
To compute the species phylogenetic tree, we aligned each of the 3,940 protein families of the persistent genome individually with the align module of PanACoTA. These alignments were concatenated to produce a large alignment matrix with 296,147 parsimony-informative sites over a total alignment of 3,740,313 bp. We then use this alignment to make the phylogenetic inference using IQ-TREE (v2.02). We used ModelFinder [134] and calculated 1,000 ultra-fast bootstrap [135]. The best-fit model was a general time-reversible model with empirical base frequencies allowing for invariable sites and discrete Gamma model with 4 rate categories (GTR+F+I+G4). We rooted the phylogenetic tree with the midpoint.root() function from the Phangorn R package [136].
We used Kaptive v2.0.0 [126,137] with default options and the “K locus primary reference” to identify the capsule locus type (CLT) of strains. Capsule regulators of BJ1, NTUH-K2044 and ST45 were detected according to a previous analysis [138]. Briefly, we compiled a dataset of proteins known to affect capsule production and performed a BLASTP search (Blast+ v2.12) [123] (default parameters) against the full proteome.
We inferred the ancestral state of each plasmid pangenome family with PastML (v1.9.23) [139] using the MAP algorithm and the F81 model.

Data analysis

All the data analyses were performed with R version 4.2 and Rstudio v2022.02.1, except linear mixed models which were computed in JMP v16 (SAS corporation) and generalized additive linear models which were computed with the mgcv R package v1.9 (function gam). For data

frame manipulations, we also used dplyr v1.0.10 along with the tidyverse packages [140] and data.table v1.12.8. We used the packages ape v5.3 [141], phangorn v2.5.5 [136], and treeio v1.10 [142] for the phylogenetic analyses. We typically show single points when there are less than ten observations, boxplots between 10-100, and violin plots when there are over 100 observations because they might reveal multi-modal underlying distributions. No statistical method was used to predetermine sample size. No data were excluded from the analyses. The Investigators were not blinded to allocation during experiments and outcome assessment.

For manuscripts utilizing custom algorithms or software that are central to the research but not yet described in published literature, software must be made available to editors and reviewers. We strongly encourage code deposition in a community repository (e.g. GitHub). See the Nature Portfolio guidelines for submitting code & software for further information.

Data

Policy information about availability of data

All manuscripts must include a data availability statement. This statement should provide the following information, where applicable:

- Accession codes, unique identifiers, or web links for publicly available datasets
- A description of any restrictions on data availability
- For clinical datasets or third party data, please ensure that the statement adheres to our policy

Source data are provided with this paper. Mutant strains, transconjugants and plasmids sequence generated in this study were deposited in the BioProject PRJNA952961. All data generated in this study have been deposited in the public repository Zenodo at: 10.5281/zenodo.10059094.
RefSeq Genome accession numbers are listed in Supplementary Table S6

Research involving human participants, their data, or biological material

Policy information about studies with human participants or human data. See also policy information about sex, gender (identity/presentation), and sexual orientation and race, ethnicity and racism.

Reporting on sex and gender	No human participant
Reporting on race, ethnicity, or other socially relevant groupings	No human participant
Population characteristics	No human participant
Recruitment	No human participant
Ethics oversight	No human participant

Note that full information on the approval of the study protocol must also be provided in the manuscript.

Field-specific reporting

Please select the one below that is the best fit for your research. If you are not sure, read the appropriate sections before making your selection.

☒ Life sciences ☐ Behavioural & social sciences ☐ Ecological, evolutionary & environmental sciences

For a reference copy of the document with all sections, see nature.com/documents/nr-reporting-summary-flat.pdf

Life sciences study design

All studies must disclose on these points even when the disclosure is negative.

Sample size	We sampled three different strains of Klebsiella pneumoniae (BJ1, NTUH, ST45); five different capsule serotypes (K1, K2, K3, K24, deltaCapsule), ten different plasmids belonging to all three types of conjugative pili present in the species (MPF-F, MPF-T, MPF-I) and performed all-vs-all combinations to measure conjugation rates. This yielded 450 conjugation experiments from the E. coli donor and 675 conjugation experiments for K.pneumoniae donors, replicated three times<br>We also sampled three different phages for which we performed all-vs-all infection matrix (n=54 pairs of phage-bacterial strain, replicated three times)<br>We sampled n=623 independent genomes of K. pneumoniae containing n=29,043 plasmids from NCBI RefSeq.
Data exclusions	No data were excluded
Replication	Experiments were repeated three times independently (biological replicates). This is the minimum number of replicates for any inferential analysis. This is the only achievable replication number given the number of experiments performed by hand (n=1200).
Randomization	Randomization was not relevant to this study (no groups).
Blinding	Blinding was not relevant to this study which relies on performing comparative genomics analyses and measuring infection rates of conjugatve plasmids which relies on exact counting of events (Colony-forming unit counting)

Reporting for specific materials, systems and methods

We require information from authors about some types of materials, experimental systems and methods used in many studies. Here, indicate whether each material, system or method listed is relevant to your study. If you are not sure if a list item applies to your research, read the appropriate section before selecting a response.

Materials & experimental systems

n/a	Involved in the study
☒	☐ Antibodies
☒	☐ Eukaryotic cell lines
☒	☐ Palaeontology and archaeology
☒	☐ Animals and other organisms
☒	☐ Clinical data
☒	☐ Dual use research of concern
☒	☐ Plants

Methods

n/a	Involved in the study
☒	☐ ChIP-seq
☒	☐ Flow cytometry
☒	☐ MRI-based neuroimaging

Plants

Seed stocks	no plant
Novel plant genotypes	no plant
Authentication	no plant
